# Terrein is an inhibitor of quorum sensing and c-di-GMP in *Pseudomonas aeruginosa*: a connection between quorum sensing and c-di-GMP

**DOI:** 10.1038/s41598-018-26974-5

**Published:** 2018-06-05

**Authors:** Bomin Kim, Ji-Su Park, Ha-Young Choi, Sang Sun Yoon, Won-Gon Kim

**Affiliations:** 1 Superbacteria Research Center, Korea Research Institute of Bioscience and Biotechnology, Yusong, Daejeon 305-806 Korea; 2 Department of Microbiology and Immunology, Yonsei University College of Medicine, Seoul, 03722 Korea

## Abstract

To address the drug-resistance of bacterial pathogens without imposing a selective survival pressure, virulence and biofilms are highly attractive targets. Here, we show that terrein, which was isolated from *Aspergillus terreus*, reduced virulence factors (elastase, pyocyanin, and rhamnolipid) and biofilm formation via antagonizing quorum sensing (QS) receptors without affecting *Pseudomonas aeruginosa* cell growth. Additionally, the effects of terrein on the production of QS signaling molecules and expression of QS-related genes were verified. Interestingly, terrein also reduced intracellular 3,5-cyclic diguanylic acid (c-di-GMP) levels by decreasing the activity of a diguanylate cyclase (DGC). Importantly, the inhibition of c-di-GMP levels by terrein was reversed by exogenous QS ligands, suggesting a regulation of c-di-GMP levels by QS; this regulation was confirmed using *P*. *aeruginosa* QS mutants. This is the first report to demonstrate a connection between QS signaling and c-di-GMP metabolism in *P*. *aeruginosa*, and terrein was identified as the first dual inhibitor of QS and c-di-GMP signaling.

## Introduction

Infections caused by multidrug-resistant (MDR) gram-negative bacteria are a growing problem^1^. There is an urgent need for new agents to treat infections caused by gram-negative bacteria that are resistant to currently available agents^2^. Traditional approaches to combat bacterial infection are aimed at killing bacteria or preventing their growth. These strategies and compounds impose substantial stress on the target bacterium, rapidly leading to resistance^3^. Interference with bacterial virulence is an emerging strategy because it applies less selective pressure for the development of resistance as most virulence traits are not essential for bacterial survival^4^. *Pseudomonas aeruginosa* is a ubiquitous gram-negative bacterium that is responsible for many opportunistic and nosocomial infections. In particular, it is fatal to cystic fibrosis patients, forming mucoid masses in lung tissue that lead to pneumonia^5^. *P*. *aeruginosa* colonizes various environmental niches by forming biofilms, which reportedly make the bacteria more resistant to antibiotics^6^. *P*. *aeruginosa* also produces various virulence factors, such as elastase, rhamnolipid, and pyocyanin. Thus, the prevention of virulence and biofilm formation may be highly attractive for the treatment of *P*. *aeruginosa*^7^.

Quorum sensing (QS) is a type of bacterial communication using diffusible molecules known as autoinducers to regulate population behaviors such as bioluminescence, the secretion of virulence factors, biofilm formation, and antibiotic resistance^8–10^. *P*. *aeruginosa* has three main QS systems: LasR-LasI, RhlR-RhlI, and PQS-PqsR^10^. LasR-LasI and RhlR-RhlI use acyl homoserine lactones (AHLs) as signaling molecules. The AHL synthases are LasI and RhlI, which produce N-(3-oxododecanoyl)-l-homoserine lactone (OdDHL) and N-butanoyl homoserine lactone (BHL), respectively. The receptors for OdDHL and BHL are the transcriptional regulators LasR and RhlR, respectively; these regulators direct the gene expression of various virulence factors and are involved in biofilm formation. In the PQS-PqsR system, 2-heptyl-3-hydroxy-4(1H) quinolone (PQS) bind to the transcriptional regulator PqsR and control the transcription of downstream targets. The relationship between QS and biofilm formation has not been fully elucidated^10^. AHL QS systems have been reported to be responsible for eDNA release by biofilms formed by the PAO1 strain of *P*. *aeruginosa*^11^ and for the production of extracellular polymeric substances by the PA14 strain^12^. Thus, *P*. *aeruginosa* QS appears to participate in the development of biofilm architecture rather than the initiation of biofilm formation.

Another important signal in bacteria that allows for adaptation to different environments is the second messenger 3,5-cyclic diguanylic acid (c-di-GMP)^13,14^. Cellular c-di-GMP controls biofilm formation, motility, and virulence as well as other behaviors, including cell cycle propagation and development, in many bacteria^13^. C-di-GMP is synthesized by diguanylate cyclase (DGC) enzymes and degraded by phosphodiesterase (PDE) enzymes. DGC/PDE enzymes contain various N-terminal sensory domains to respond to environmental signals, including oxygen, light, nitric oxide, and specific compounds^15^. Binding of c-di-GMP with the transcription factor FleQ leads to the down-regulation of flagellar gene expression and the up-regulation of exopolysaccharide gene expression to initiate biofilm formation. C-di-GMP also plays a role in the maturation and dispersal of biofilms. Thus, c-di-GMP is emerging as an attractive target for clinical intervention^16^.

**Figure 1:**
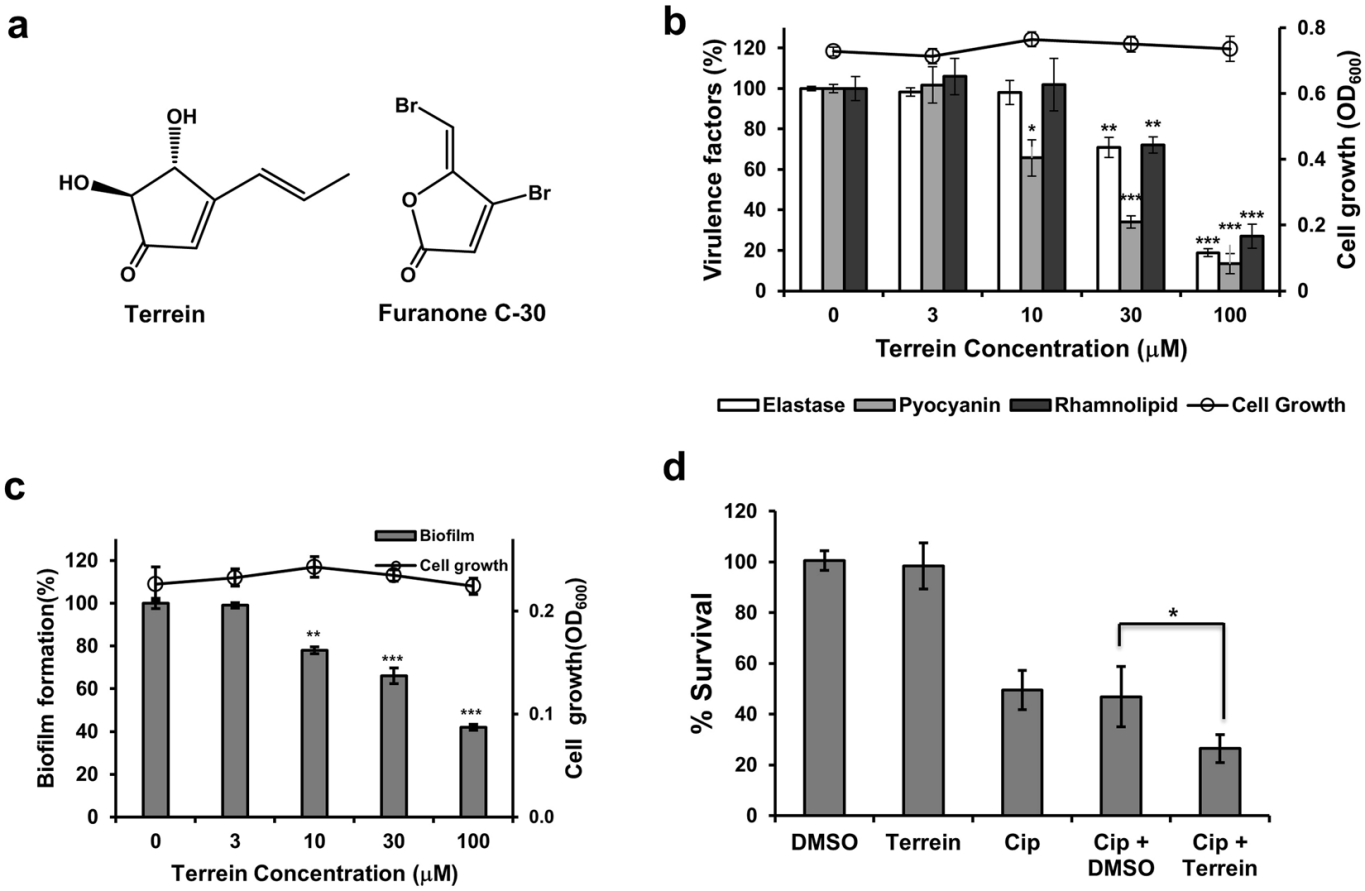
Effects of terrein on virulence factor production and biofilm formation. (**a**) Chemical structures of terrein and furanone C-30. (**b**) Effects of terrein on virulence factor production and cell viability in *P*. *aeruginosa* PAO1. PAO1 cells were grown in LB medium containing various concentrations of terrein for 24 h, followed by the measurement of cell density at 600 nm, elastase activity, pyocyanin, and rhamnolipid in the culture supernatants. (**c**) Effects of terrein on biofilm formation and cell viability in *P*. *aeruginosa*. *P*. *aeruginosa* biofilms formed in the presence of terrein for 9 h. The biofilm cells attached to the well surface were assayed using crystal violet staining. (**d**) Effects of terrein on the antibiotic tolerance of biofilms. Preformed biofilms (6-h) of PAO1 cells were treated with ciprofloxacin (Cip) alone or together with DMSO or 300 μM terrein for 3 h. The biofilms were dissociated from the wells by gentle sonication, and the bacteria were enumerated by plating. Three independent experiments were performed in triplicate, and the mean ± standard deviation (SD) values are displayed in each bar. *P < 0.01; **P < 0.001; ***P < 0.0001 versus untreated cells.

While screening for an inhibitor of virulence factors and biofilms in *P*. *aeruginosa* from a microbial metabolites library, we identified terrein from a fermentation culture of the FN423 fungal strain (Fig. 1a). Terrein is a five-membered ketone ring, that is more stable than known QS inhibitors, such as furanone C-30, which contains a lactone skeleton^17^. Terrein reduced the production of virulence factors, such as elastase, pyocyanin, and rhamnolipid, as well as biofilm formation without bactericidal action. Terrein inhibited both QS and c-di-GMP signaling. Here, we report the *in vitro* and *in vivo* anti-virulence and anti-biofilm activity of terrein against *P*. *aeruginosa*, as well as its inhibition of QS and c-di-GMP and connections between QS and c-di-GMP in *P*. *aeruginosa*.

## Results

### Screening and isolation of a fungal strain-derived inhibitor of virulence factor production

To identify an inhibitor of virulence factor production, over 12,300 microbial extracts were screened and evaluated for their ability to inhibit the production of elastase by *P*. *aeruginosa* PAO1. This procedure led to the selection of two strains. One of them, the fungal strain FN423, was identified as *Aspergillus terreus* using phylogenetic analysis based on 18S rDNA. The active component was isolated via bioassay-guided fractionation of the culture supernatants (Supporting information) and then identified as terrein^18^, 4,5-dihydroxy-3-propenylcyclopentenone, with a molecular weight of 154 by mass and NMR spectral analysis together with its specific rotation value (Figs 1a and S1b and c).

### Anti-virulence activity of terrein against *P*. *aeruginosa* PAO1

The inhibitory activity of terrein against the production of virulence factors, such as elastase, pyocyanin, and rhamnolipid, by PAO1 cells was evaluated. First, the antibacterial activity of terrein was examined in the same LB broth used for the virulence factor assays using the viable cell count and optical density assays. Terrein did not affect the viability of the PAO1 cells up to 100 μM (Fig. S2a).

Terrein prevented elastase production in PAO1 cells in a dose-dependent manner (Fig. 1b). The elastase activity of the PAO1 cells in the presence of 30 and 100 μM terrein for 24 h was significantly inhibited by 29.1% and 81.1%, respectively, compared with that of the untreated cells. Inhibition of elastase activity by terrein without any associated effects on cell viability was more clearly demonstrated in a growth-dependent assay (Fig. S2b and c). Next, we investigated whether the terrein-induced reduction in elastase activity was mediated by the suppressed production of elastase or by the direct inhibition of elastase activity. Terrein did not inhibit elastase activity, but it did inhibit elastase production (Fig. S2d). However, the diacetyl derivative of terrein (Fig. S3a) demonstrated no activity (Fig. S3b), suggesting a critical role of the hydroxyl groups in terrein activity.

Pyocyanin and rhamnolipid are also important virulence factors for infection by *P*. *aeruginosa*^19,20^. Pyocyanin production by PAO1 cells in the presence of 10, 30, and 100 μM terrein for 24 h was significantly inhibited by 34.3%, 65.9% and 86.6%, respectively, compared with the untreated cells, and no effect on cell viability was observed, as expected (Fig. 1b). Similarly, rhamnolipid production by PAO1 cells was also inhibited by 28.0% and 73.1% in the presence of 30 and 100 μM terrein, respectively (Fig. 1b).

### Anti-biofilm activity of terrein against *P*. *aeruginosa* PAO1

Rhamnolipid and pyocyanin have been reported to be important for biofilm formation in *P*. *aeruginosa* PAO1^21,22^. Since terrein inhibited the production of rhamnolipid and pyocyanin, the effect of terrein on the development of growing *P*. *aeruginosa* PAO1 biofilms was investigated by staining the biofilm biomass. Biofilm formation was significantly reduced by adding 10–100 μM terrein, while cell growth was not affected, which showed the same activity as the positive control, furanone C-30 (Fig. S4a). Since the structure of terrein, a ketone ring compound, has been suggested to demonstrate greater chemical stability than furanone C-30, a lactone-ring compound, the anti-biofilm activities of both compounds were compared with different incubation times. Terrein maintained its anti-biofilm activity after treatment for 24 h, while furanone C-30 completely lost its activity (Fig. S4b).

The development of a biofilm allows aggregated cells to become increasingly resistant to antibiotics^23^. To determine whether terrein reduces the antibiotic resistance of biofilms, the effects of terrein on the antibiotic tolerance of preformed biofilms were examined. A 6-h PAO1 biofilm demonstrated tolerance to ciprofloxacin (Fig. 1d). However, combined treatment of terrein with ciprofloxacin significantly enhanced the killing effects of ciprofloxacin, while terrein and DMSO showed no killing effect.

The anti-biofilm activity of terrein, as described above, was supported by confocal laser scanning microscopy. When PAO1 cells were statically grown for 6 h on glass coverslips, a biofilm formed with a thickness of 18.9 μm. However, when cells were grown in the presence of 100 μM terrein, the biofilm thickness was substantially decreased to 10.1 μm (Fig. S5).

### Inhibition of *P*. *aeruginosa* virulence toward *Caenorhabditis elegans* by terrein

**Figure 2:**
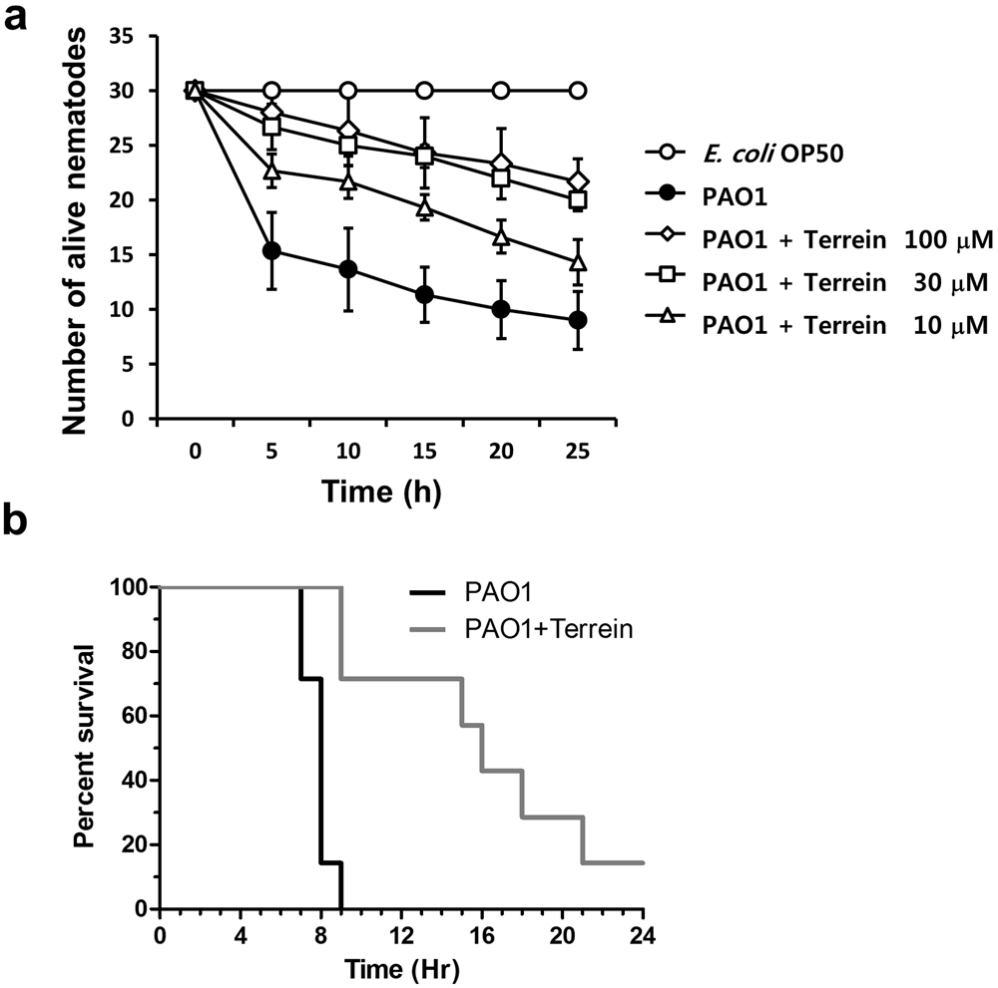
*In vivo* anti-virulence activity of terrein. (**a**) Inhibition of PAO1 virulence by terrein toward *C*. *elegans*. *C*. *elegans* was applied to lawns of *E*. *coli* OP50 (open circles) or PAO1 (filled circles) on plates containing different concentrations of terrein. The percentage of live nematodes was calculated every 5 h for 30 h. Three independent experiments were performed in triplicate, and the mean ± SD values are displayed in each graph. The presence of terrein (10~100 μM) significantly protected *C*. *elegans* from killing (*P* < 0.0001). (**b**) Inhibition of *P*. *aeruginosa* PAO1 virulence by terrein in a murine airway infection model. Mouse survival rate following infection with PAO1 without (black) or with (gray) terrein (100 μM). Seven mice were used in each group, and the infection dose was 1 × 10^7^ cfu cells.

To determine if terrein prevents the *in vivo* virulence of *P*. *aeruginosa*, a *C*. *elegans* fast-kill infection assay was performed. Feeding with *P*. *aeruginosa* PAO1 rapidly caused the death of *C*. *elegans*, as evidenced by the death of 80% of nematodes after 30 h (Fig. 2a). Indeed, treatment with terrein prevented *C*. *elegans* killing in a dose-dependent manner. The percentage of nematode death significantly decreased in the presence of terrein at 10–100 μM. These results clearly indicated that terrein prevented *P*. *aeruginosa* infection of *C*. *elegans*.

### *In vivo* effectiveness of terrein against *P*. *aeruginosa* in a murine airway infection model

To further assess the protective effect of terrein against PAO1 infection, an *in vivo* infection experiment using a murine airway infection model was conducted. A total of seven mice were used in each group for a survival experiment. PAO1-infected mice started to expire at 7 h post-infection, and an additional 2 h was required before complete expiration. However, mice treated with terrein (100 μM) at the time of infection started to expire at 9 h post-infection. Six of the seven mice treated with terrein expired gradually until termination of the experiment at 24 h (Fig. 2b), strongly suggesting the presence of terrein-mediated virulence attenuation in the mouse airway. The bacterial loads recovered in mouse lungs were lower in the terrein-treated group (9.33 × 10^7^ cfu/mL versus 2.86 × 10^8^ cfu/mL), demonstrating that terrein treatment resulted in decreased bacterial colonization at an early stage of infection (Fig. S6).

### Terrein-mediated inhibition of QS

QS, a cell density-based intercellular communication system, plays a key role in the regulation of bacterial virulence and biofilm formation^24^. To ascertain whether terrein blocks QS receptors, an AHL-based *in vitro* QS competition assay was carried out using the two reporter strains, *Chromobacterium violaceum* CV026 and *Agrobacterium tumefaciens* NT1^25^. *A*. *tumefaciens* NT1 contains a *lacZ* reporter gene fused to the gene encoding the TraR receptor, which detects AHLs that have a long carbon chain, such as OdDHL, leading to the production of a cyan color^26^. The *C*. *violaceum* CV026 strain was constructed to express the CviR receptor, which senses exogenous AHLs, such as BHL with short carbon chain lengths, resulting in the production of a purple pigment called violacein^27^. Since the TraR and CviR receptors in *A*. *tumefaciens* NT1 and *C*. *violaceum* CV026 are homologs of LasR and RhlR receptors, respectively, in *P*. *aeruginosa*, these reporter strains are frequently used to conduct a competitive binding assays for the LasR and RhlR receptors in *P*. *aeruginosa*^28^.

**Figure 3:**
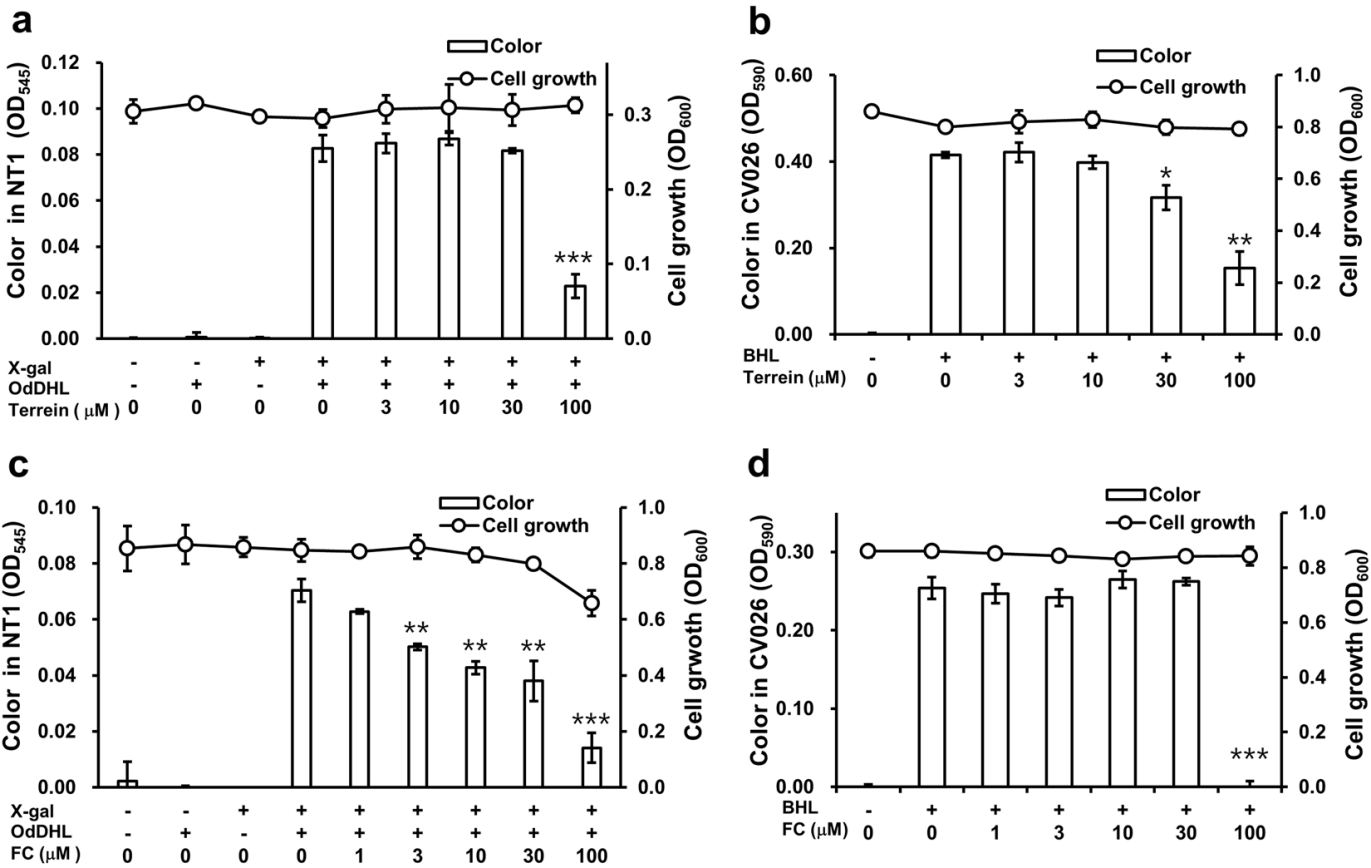
QS inhibition by terrein. The reporter strains and *A*. *tumefaciens* NT1 and *C*. *violaceum* CV026 were used to assay QS competition related to BHL and OdDHL, respectively. *A*. *tumefaciens* NT1 and *C*. *violaceum* CV026 were treated with 1 μM OdDHL and 500 μM BHL, respectively, and grown in the presence of different concentrations of terrein or furanone C-30 (a positive control) for 24 h. After cell viability was measured at 600 nm, color changes were measured. (**a**) Terrein – OdDHL competition. (**b**) Terrein – BHL competition. (**c**) Furanone C-30 – OdDHL competition. (**d**) Furanone C-30 – BHL competition. Three independent experiments were performed in triplicate, and the mean ± SD values are displayed in each bar. *P < 0.01; **P < 0.001; ***P < 0.0001 versus untreated cells.

NT1 cultures produced no pigment in the absence of OdDHL, but pigment was produced when OdDHL was added, as expected. Pigment production decreased by 72.3% when cultures were treated with terrein at 100 μM for 24 h, with no associated effect on cell growth (Fig. 3a). Similarly, production of the violacein pigment was decreased by terrein in CV026 cultures. Inhibition of up to 30.9% and 61.3% was observed when the cultures were treated with terrein at 30 and 100 μM, respectively (Fig. 3b). This finding indicated that terrein antagonistically inhibited the bindings of OdDHL and BHL to the cognate receptors TraR and CviR, respectively. Furanone C-30, a well-known QS inhibitor, antagonized the binding of OdDHL to TraR at 3–100 μM as a positive control (Fig. 3c), showing antibacterial activity against NT1 cells at 100 μM, whereas antagonized CviR only at 100 μM, (Fig. 3d). This result indicated a higher antagonistic activity of furanone C-30 to LasR rather than RhlR as reported previously^29^.

### Inhibitory activity of terrein on production of QS signaling molecules and expression of QS-regulated genes

**Figure 4:**
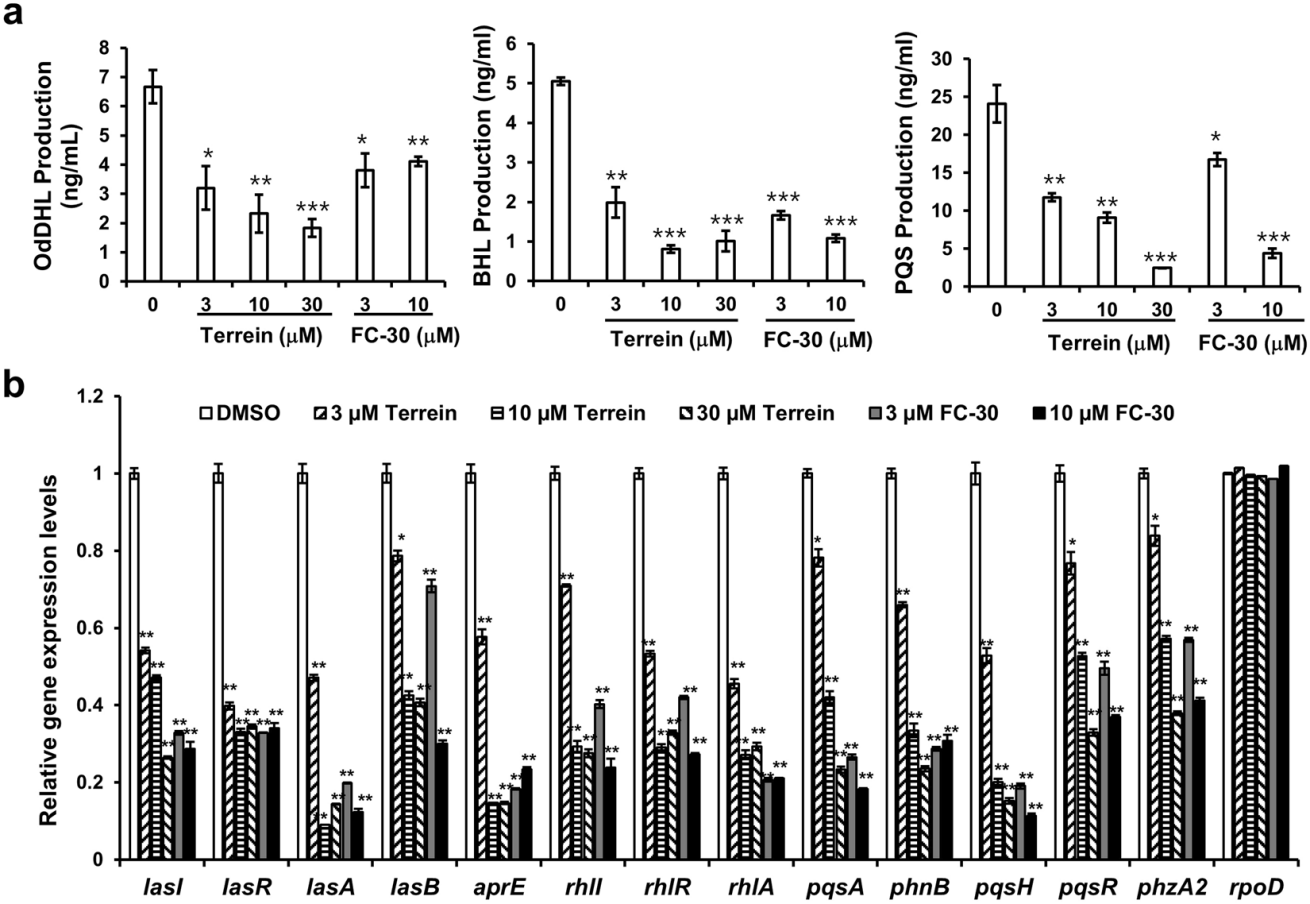
Effects of terrein on QS signaling molecule production and QS gene expression. PAO1 were cultured in LB medium containing different terrein or furanone C-30 concentrations for 24 h. (**a**) Effect of terrein on the three main QS molecules, OdDHL, BHL, and PQS. The QS molecules were extracted from the culture supernatants and quantitatively analyzed by LC-MS/MS. The experiment shown is representative of three independent experiments in triplicate, and the mean ± SD values are displayed in each bar. *P < 0.01; **P < 0.001; ***P < 0.0001 versus DMSO treatment. (**b**) Effect of terrein on the expression of QS-regulated genes as assessed by RT-qPCR. The experiment shown is representative of three independent experiments in triplicate, and the mean ± SD values are displayed in each bar. *P < 0.001; **P < 0.0001 versus DMSO treatment.

To determine whether terrein also affects QS signal production in PAO1 strains, production of AHL and PQS molecules was quantitatively determined by LC-MS/MS (Fig. 4a). The untreated PAO1 cultures yielded 6.67 ± 0.57 ng/mL, 5.06 ± 0.10 ng/mL, and 24.1 ± 2.5 ng/mL for OdDHL, BHL, and PQS, respectively, in the 24-h culture supernatants. Production of the three main QS molecules was dose-dependently inhibited by terrein at 3–30 μM. As a positive control, furanone C-30 also reduced production at 3 and 10 μM.

The effect of terrein on the transcription of QS-regulatory genes (*lasI*, *IasR*, *rhlI*, *rhlR*, *pqsA*, *phnB*, *pqsH*, and *pqsR*) and key QS-controlled genes (*lasA*, *lasB*, *aprE*, *rhlA*, and *phzA2*) in PAO1 strains was measured by real-time quantitative PCR (RT-qPCR) (Fig. 4b). The transcriptional levels of the QS-regulatory genes were dose-dependently repressed by terrein at 3–30 μM. Terrein also reduced the expression of the QS-controlled virulence factors, including exoprotease, rhamnolipid, and pyocyanin.

### Reduction of cellular c-di-GMP levels by terrein

**Figure 5:**
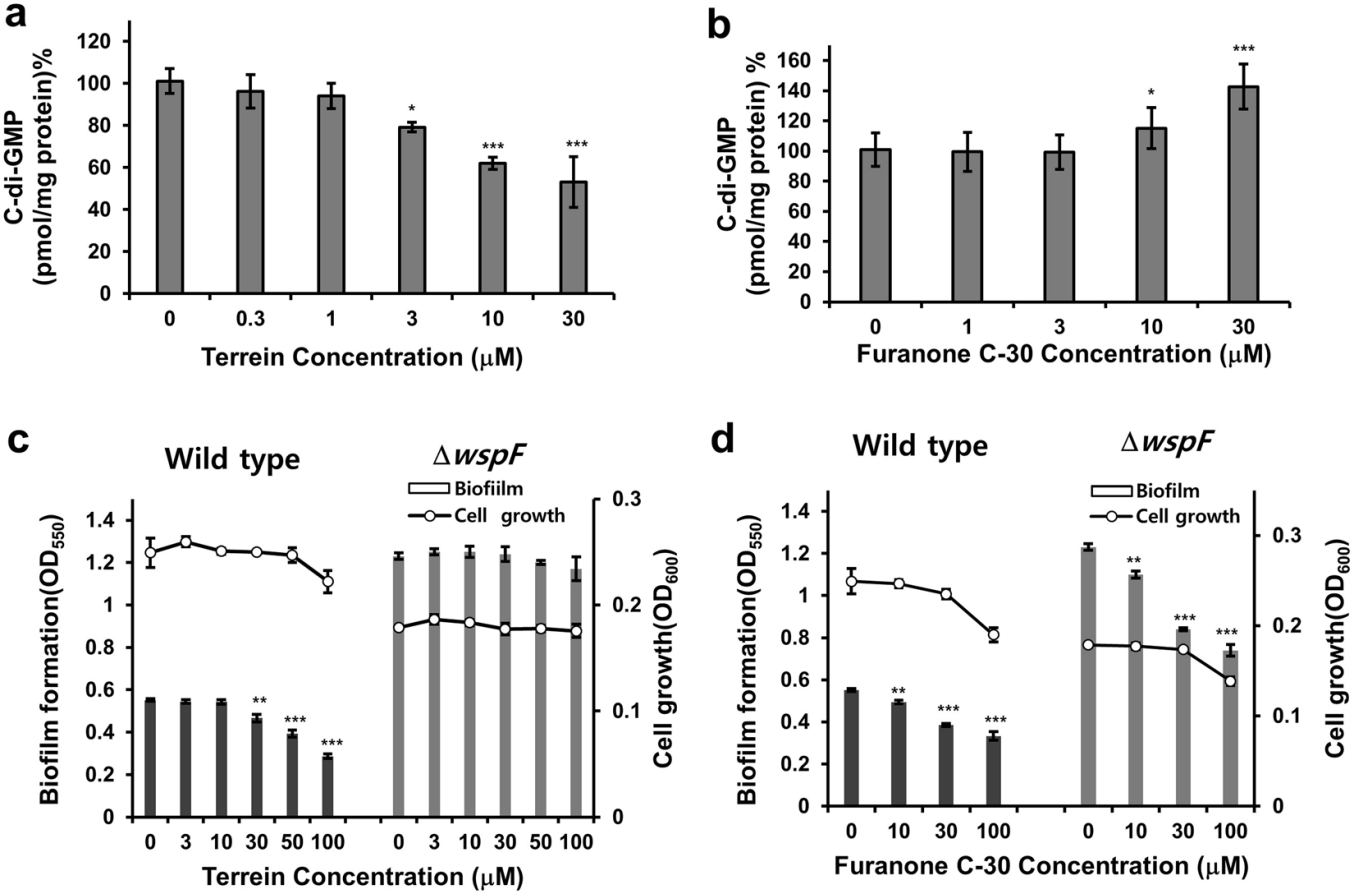
Effects of terrein on cellular c-di-GMP. (**a** and **b**) Cellular c-di-GMP levels in biofilm cells of PAO1 cultured with different terrein or furanone C-30 concentrations for 9 h. After the biofilms were dissociated from the wells by gentle sonication, cellular c-di-GMP was extracted from the biofilm cells, measured, and normalized to the total proteins. (**c** and **d**) Comparison of biofilm formation by the *P*. *aeruginosa* wspF mutant versus wild-type PA14 cells cultured with different terrein or furanone C-30 concentrations for 9 h. Three independent experiments were performed in triplicate, and the mean ± SD values are displayed in each bar. *P < 0.01; **P < 0.001; ***P < 0.0001 versus DMSO treatment.

Cellular c-di-GMP plays important roles in the transition from planktonic to biofilm lifestyles, the regulation of motility, and the induction of related virulence factors^13^. Possible inhibition of cellular c-di-GMP levels by terrein was investigated in PAO1 biofilm cells. Cellular c-di-GMP levels were reduced by terrein at 3–30 μM (Figs 5a and S7a). In contrast, furanone C-30 increased cellular c-di-GMP levels at 10 and 30 μM without affecting cell growth (Figs 5b and S7b).

To validate that the inhibitory activity exerted by terrein on biofilm formation is due to the inhibition of c-di-GMP production, the anti-biofilm activity of terrein in a c-di-GMP-overproducing mutant (Δ*wspF*)^30^ and a PA14 wild-type strain was examined. First, effects of terrein on virulence factors, c-di-GMP levels, QS signal production, and QS gene expression in the PA14 strain were investigated. Terrein inhibited the production of virulence factors, QS signaling molecules, and QS gene expression with similar activity in PA14 cells to that in PAO1 cells, while it reduced c-di-GMP levels with weaker activity in PA14 cells than in PAO1 cells (Figs S8 and S9). The WspF protein, a methyltransferase, has been reported to control the activity of the WspR protein, which contains a CheY-like receiver and a DGC (GGDEF) domain. Since Δ*wspF* constitutively activates the WspR protein, Δ*wspF* causes an elevation of cellular c-di-GMP levels and biofilm formation^30^. Thus, if terrein inhibits biofilm via reduction of the intracellular c-di-GMP, terrein would inhibit biofilm formation in the c-di-GMP-overproducing mutant less than in the wild type. Δ*wspF* induced 2.2-fold higher biofilm formation compared to the wild-type strain as expected (Fig. 5c). Indeed, terrein did not inhibit biofilm formation in the c-di-GMP-overproducing mutant (Δ*wspF*) up to 100 μM, while it inhibited biofilm formation in the PA14 wild-type strain at 30–100 μM (Fig. 5c). Consistently, terrein did not reduce the intracellular c-di-GMP levels in the Δ*wspF* strain up to 100 μM (Fig. S10). In contrast, furanone C-30 showed almost the same inhibitory activity on biofilm formation at 10–100 μM in both Δ*wspF* and the wild-type strain, as expected (Fig. 5d). This indicated that terrein inhibited biofilm formation by decreasing cellular c-di-GMP levels.

**Figure 6:**
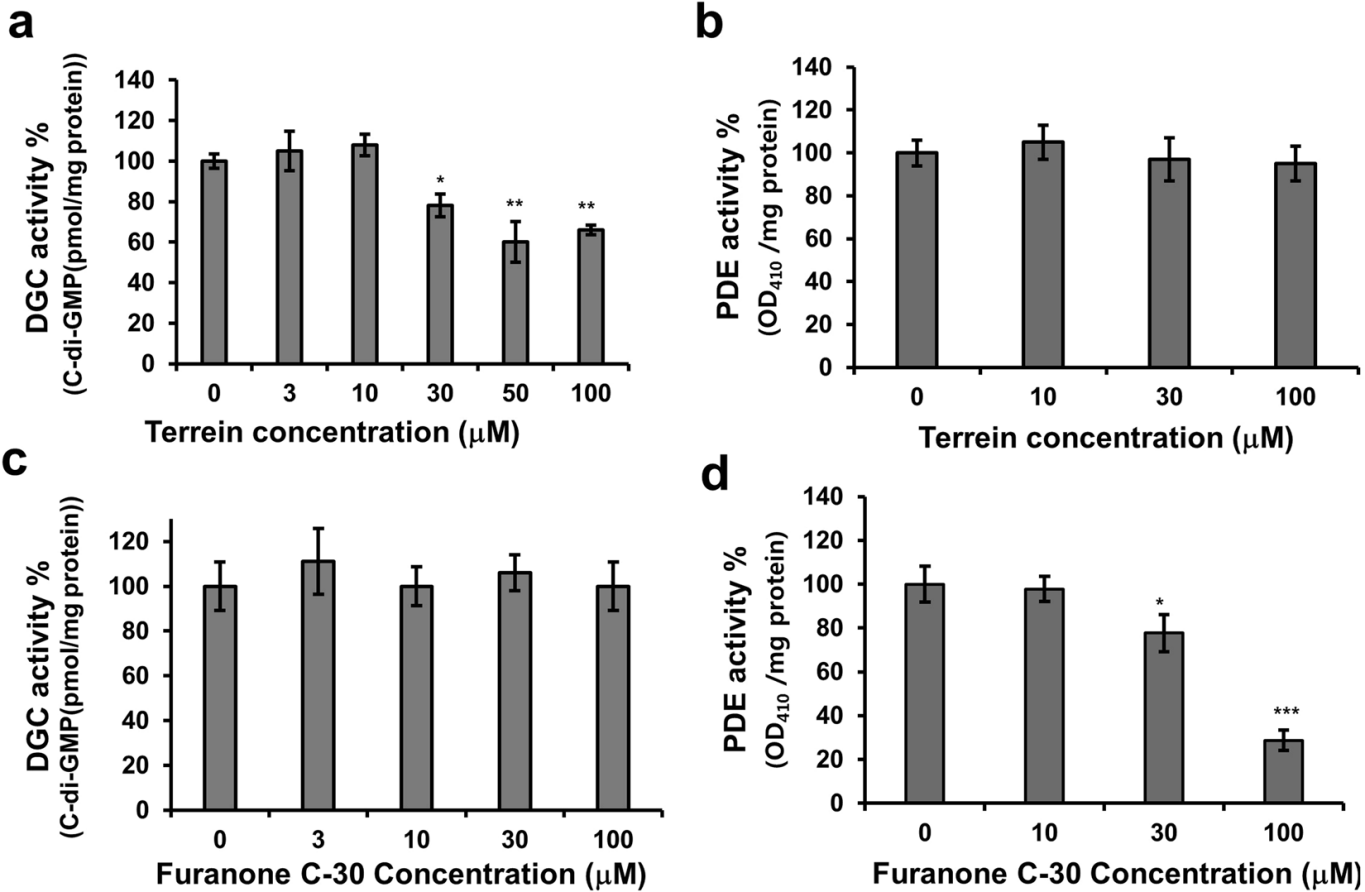
Effects of terrein on DGC and PDE activity. (**a** and **c**) DGC and (**b** and **d**) PDE activity in PA14 cells cultured with terrein or furanone C-30 for 6 h. Three independent experiments were performed in triplicate, and the mean ± SD values are displayed in each bar. *P < 0.001; **P < 0.0001; ***P < 0.00001 versus untreated cells.

C-di-GMP has been reported to be biosynthesized by DGC and degraded by PDE^31^. The activity of these two enzymes in PA14 cells grown with terrein was examined to test whether terrein inhibited cellular c-di-GMP levels, either via decreased DGC activity or the enhanced PDE activity. For validation of the DGC assay, the levels of the natural c-di-GMP in the cell lysates was first analyzed. The natural c-di-GMP levels did not change in the DGC assay without GTP addition as a substrate, whereas they increased in the presence of GTP (Fig. S11a). These findings suggested that the c-di-GMP levels in the DGC assay could be controlled by only the DGC enzyme and not by PDE. For the PDE assay, a PDE-specific substrate, bis-p-nitrophenol phosphate (bis-pNPP), was used. Indeed, DGC activity decreased in PA14 grown with terrein at 30–100 μM (Figs 6a and S11b), whereas the degradation of bis-pNPP did not change, even at 100 μM (Figs 6b and S11c). In contrast, furanone C-30-treated PA14 showed a decrease in activity of c-di-GMP-specific PDE at 30 and 100 μM (Fig. 6d), which accounted for the enhanced activity of furanone C-30 in cellular c-di-GMP production, while DGC activity did not change at 100 μM (Fig. 6c).

### The reversion of terrein-induced decreases in c-di-GMP levels by exogenous QS ligands

**Figure 7:**
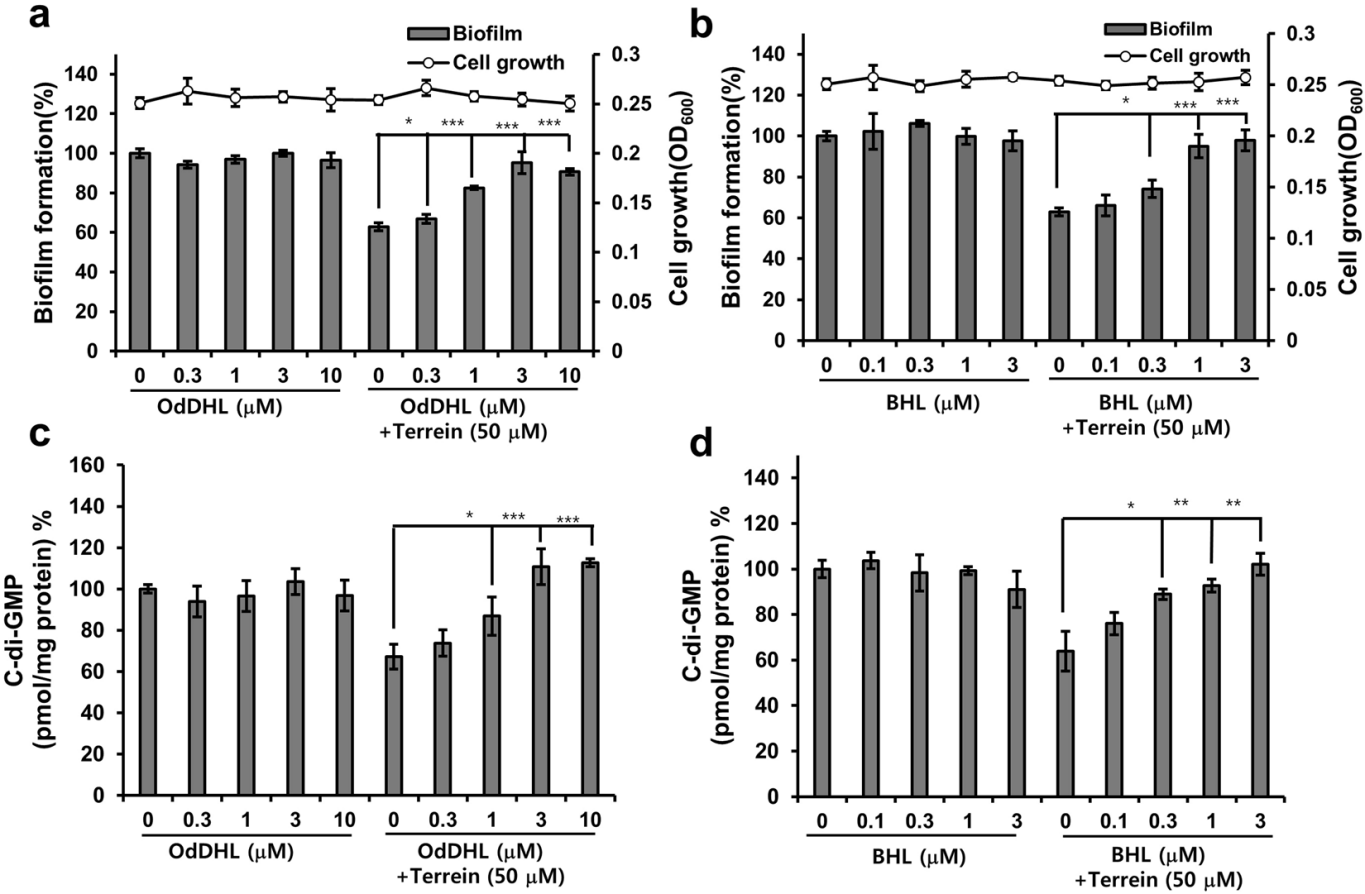
Effects of exogenous QS ligands on the inhibition of biofilm and cellular c-di-GMP by terrein. (**a** and **b**) Biofilm formation and (**c** and **d**) c-di-GMP concentrations of PA14 cells cultured with terrein (50 μM) in the presence or absence of a different concentration of OdDHL or BHL for 9 h. Three independent experiments were performed in triplicate, and the mean ± SD values are displayed in each bar. *P < 0.01; **P < 0.001; ***P < 0.0001 versus terrein-treated cells.

Interestingly, terrein inhibited both QS and c-di-GMP signaling. The connection between QS and c-di-GMP is poorly understood in *P aeruginosa*. To determine whether terrein inhibited cellular c-di-GMP levels via QS signaling, the effects of two exogenous QS ligands, OdDHL and BHL, on the terrein-induced reduction of cellular c-di-GMP concentrations were investigated. First, the effects of OdDHL and BHL on the terrein-induced inhibition of PA14 biofilm cells were tested. Exogenous supplementation of both OdDHL (0.3–10 μM) and BHL (0.1–3 μM) reversed the biofilm inhibitory activity of terrein at 50 μM in a dose-dependent manner (Fig. 7a and b). Additionally, supplementation of both OdDHL and BHL at the same concentration reversed the inhibitory activity of terrein on cellular c-di-GMP production (Figs 7c,d and S12a and b). This finding suggested that terrein decreased cellular c-di-GMP levels via QS signaling and that there might be a strong relationship between QS and c-di-GMP production.

### Relationship between QS and c-di-GMP

**Figure 8:**
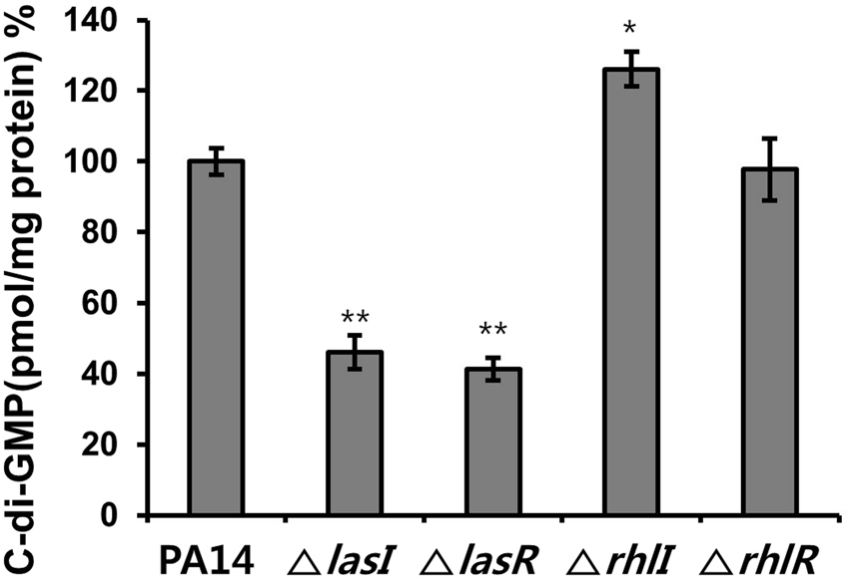
C-di-GMP levels in *P*. *aeruginosa* QS mutants. The cellular c-di-GMP concentration in biofilm cells of *P*. *aeruginosa* QS mutants (*lasI*, *lasR*, *rhlI*, *rhlR* mutants) cultured for 9 h was compared with those in wild-type PA14. Three independent experiments were performed in triplicate, and the mean ± SD values are displayed in each bar. *P < 0.001; **P < 0.0001 versus wild-type PA14.

To demonstrate that cellular c-di-GMP levels in biofilm cells are regulated by QS in *P*. *aeruginosa*, c-di-GMP levels in the biofilm cells of QS-related PA14 mutants (Δ*lasI*, Δ*lasR*, Δ*rhlI*, and Δ*rhlR*)^32^ were analyzed. As shown in Figs 8 and S13a, c-di-GMP levels were reduced by 53.9% in the Δ*lasI* mutant compared with wild-type PA14 cells, while they increased by 26.1% in the Δ*rhlI* mutant. Additionally, c-di-GMP levels decreased in the Δ*lasR* mutant as much as in the Δ*lasI* mutant, but they did not change in the Δ*rhlR* mutant. These results clearly indicate that Las-QS positively regulated c-di-GMP levels via LasR, but Rhl-QS negatively regulated c-di-GMP levels via targets other than RhlR. This regulation of c-di-GMP levels by QS was supported by the effects of exogenous QS ligands on c-di-GMP levels of the Δ*lasI* and Δ*rhlI* mutants (Fig. S14). The addition of OdDHL increased the intracellular c-di-GMP levels in both mutants, whereas BHL decreased these levels, as expected. Additionally, terrein increased c-di-GMP levels in the Δ*lasI* mutant, whereas it decreased these levels in the Δ *rhlI* mutant, as expected (Fig. S14). The terrein-induced increases in c-di-GMP levels of the Δ*lasI* mutant was reversed by addition of OdDHL (Fig. S14a), which is consistent with the results in Fig. 7c and d. These results demonstrate that the QS system regulates cellular c-di-GMP levels in *P*. *aeruginosa*.

## Discussion

Terrein inhibited the production of virulence factors such as elastase, pyocyanin, and rhamnolipid, as well as biofilm formation in *P*. *aeruginosa* PAO1 and PA14 strains. In addition, terrein showed *in vivo* anti-virulence effect in *C*. *elegans* and mice. Terrein blocked QS receptors, such as furanone C-30, in a QS competition assay using reporter strains. However, notably, terrein antagonized both LasR and RhlR with almost the same potency, while furanone C-30 antagonized LasR more potently than RhlR. Additionally, terrein inhibited the production of QS signaling molecules and the expression of QS-regulated genes in PAO1 and PA14 strains. Interestingly, terrein also decreased cellular c-di-GMP levels in PAO1 and PA14 strains, while furanone C-30 increased these levels. Since cellular c-di-GMP is known to regulate biofilm formation, it was suggested that terrein inhibited biofilm formation via the inhibition of c-di-GMP production. This hypothesis was confirmed using a *P*. *aeruginosa* mutant (Δ*wspF*) that overproduces cellular c-di-GMP. Reversion of the c-di-GMP inhibitory activity of terrein by exogenous QS ligands suggested that there may be a relationship between QS and c-di-GMP signaling. The connection between QS and c-di-GMP was confirmed using the QS mutants of PA14. This is the first report to clearly demonstrate the connection between QS and c-di-GMP in *P*. *aeruginosa*.

C-di-GMP is synthesized from two GTP molecules by DGC enzymes containing GGDEF domains and is degraded by PDE enzymes containing EAL or HD-GYP domains^13^. Unlike QS, c-di-GMP signaling employs multiple signaling pathways, and many bacteria encode a wide array of c-di-GMP synthesis and degradation proteins with sensory domains that respond to environmental signals^13^. *E*. *coli* has 12 DGC proteins, 10 PDE proteins, and 7 proteins that have both DGC and PDE domains. *P*. *aeruginosa* PA14 has 40 c-di-GMP metabolic proteins^33^. To test whether QS systems control c-di-GMP levels via the regulation of c-di-GMP metabolic enzymes DGC and PDE, the activity of these two enzymes in the QS mutants was examined (Fig. S13b and c). Although total DGC and PDE activities in response to terrein in QS mutants were determined, consistent with the c-di-GMP levels in the QS mutants (Fig. 8), DGC activity was reduced by 45.0% and 39.2% in the Δ*lasI* and Δ*lasR* mutants, respectively, compared with wild type, whereas DGC activity was not affected in the Δ*rhlI* and Δ*rhlR* mutants (Fig. S13b). This finding suggested that Las-QS might positively regulate DGC via LasR, whereas Rhl-QS did not. In contrast, the activity of c-di-GMP-specific PDE was reduced by 53.1% in the Δ*rhlI* mutant only (Fig. S13c), in agreement with the c-di-GMP levels in the QS mutants (Fig. 8). Thus, Rhl-QS might positively regulate PDE via targets other than RhlR, whereas Las-QS did not. Taken together, these results suggested that Las-QS might positively regulate c-di-GMP levels by inducing DGC activity, whereas Rhl-QS might negatively regulate c-di-GMP levels by inducing PDE activity.

As both QS and c-di-GMP regulate virulence and biofilm formation^34^, it is presumed that the two signaling pathways may be linked and/or intersect. The linkage between QS and c-di-GMP has been investigated in some bacteria^15,31^. In *Vibrio cholerae*, binding of the QS autoinducers AI-1 and AI-2 and their receptors leads to the expression of HapR (a LuxR homolog), which in turn represses expression of the transcription factors VpsT and AphA. VpsT binds to c-di-GMP to increase the expression of several genes that are required for biofilm development^35^, while AphA induces virulence gene expression and represses the expression of AcgA (a PDE) and AcgB (a DGC)^36^. In *Xanthomonas campestris*, a diffusible signaling factor (DSF) modulates the QS system, and the binding of DSF to its receptor, RpfC, activates the response regulator RpfG, which functions as a PDE to degrade c-di-GMP^37^. However, the connection between QS and c-di-GMP signaling has been poorly understood in *P*. *aeruginosa*. In PA14, Wood reported that a tyrosine phosphatase, TpbA, inhibits the activity of TpbB (a DGC) by dephosphorylation to reduce biofilm formation, and Las-QS positively regulates the expression of TpbA^38^, whereas Rhl-QS does not. TpbA also leads to increased *rhl* transcription. Therefore, this study implies that QS negatively affects cellular c-di-GMP production in *P*. *aeruginosa*; however, c-di-GMP production under different QS states has not yet been determined^15^.

Terrein was first isolated from *A*. *terreus* as a secondary bioactive metabolite in 1935^17^. Terrein has been reported to possess certain biological activities, such as anti-melanogenesis^39^, an anti-proliferative effect in human cancer cells^40^ and an anti-inflammatory effect in human gingival fibroblasts^18^. Mechanistically, terrein has been reported to show anti-cancer activity in human cancer cells by inducing apoptosis and cell cycle arrest^40,41^, and, to my knowledge, a general toxicity of terrein has not been reported. However, the anti-virulence activity of terrein against bacteria is reported for the first time in this study.

Since inhibition of QS has been regarded as an attractive approach to control bacterial infection^7,42^, previous screens have identified small-molecule inhibitors of *P*. *aeruginosa* QS receptors, but very few inhibitors, such as furanone C-30, have been shown to influence virulence in animal models^43–47^. However, furanone C-30 contains a lactone moiety that is sensitive to chemical and enzymatic hydrolysis, and many efforts to develop non-lactone or thiolactone QS inhibitors have been undertaken^48,49^. Terrein not only showed *in vivo* anti-virulence effects in *C*. *elegans* and mouse models, but it also contains a stable ketone ring moiety. Thus, regarding its application as a drug candidate, terrein possess structural strength. Further optimization using a previously reported method for the synthesis of terrein and its analogs may improve efficacy^50^. Thus, terrein has great potential for development as a new anti-virulence drug against multidrug-resistant bacterial pathogens.

## Methods

### Elastase assay

The production of elastase in *P*. *aeruginosa* PAO1 was assayed using an Elastin-Congo red assay as previously described^51^. *P*. *aeruginosa* PAO1 was cultivated in LB medium overnight at 220 rpm at 37 °C. Overnight cultures were diluted 100-fold in the same medium and dispensed at 0.1 mL/well in a 96-well microtiter plate. Microbial extracts, test compounds dissolved in DMSO, or DMSO as a negative control were added to the PAO1 cultures. After incubation for 12 h at 37 °C, cell viability was assayed by either measuring the optical density (OD) at 600 nm or counting viable cells, which were then centrifuged at 4000 rpm for 10 min. The elastase activity of the culture supernatants was measured using Elastin-Congo Red as an elastase substrate.

### Pyocyanin, rhamnolipid, and biofilm assay

The production of pyocyanin^52^ and rhamnolipid^53^ in *P*. *aeruginosa* PAO1 was assayed as previously described. *P*. *aeruginosa* biofilms were assayed in a 96-well polystyrene microtiter plate as previously described (Supporting Information)^54^. After cell viability was assayed by measuring the OD at 600 nm, extracted pyocyanin and rhamnolipid were measured at 520 and 421 nm, respectively, and biofilm cells attached to the well surface were assayed using crystal violet staining. For the biofilm assay, biofilms were grown in a 96-well plate for 6 h. After planktonic cells in each well were discarded, the attached biofilms were treated with 0.01 mg/L ciprofloxacin (Sigma) combined with DMSO or terrein for 3 h. Bacteria inside the biofilm were detached by sonication at a frequency of 40 KHz with a power output of 50 W for 2 min. Viable cells were enumerated by serial dilution and plating.

### *C*. *elegans* life span assay

A *C*. *elegans* killing assay was performed as previously described^44^. *C*. *elegans* was propagated on NGM plates with a lawn of *E*. *coli* OP50 at 20 °C for 48 h to reach the L4 stage. Killing plates were prepared by spreading 5 μL of the overnight-cultured PAO1 cells on a 35-mm petri plate containing 4 mL of PGS agar. Control plates were prepared by spreading *E*. *coli* OP50 instead of PAO1. Plates were incubated at 37 °C for 24 h and then transferred to 23 °C for 24 h. Thirty L4 stage worms were placed on the plates and incubated at room temperature. Worms were scored for survival every 5 h for 30 h. Test compounds dissolved in DMSO or DMSO as a negative control were added to the killing plates.

### Murine airway infection

To test the effect of terrein *in vivo*, twenty 5-week-old BALB/C inbred female mice (Orient, Korea) were infected with 1 × 10^7^ PAO1. Bacteria that had been precultured for 16 h were diluted in fresh LB medium (1:100) and grown until they had reached an OD_600_ corresponding to 1 × 10^9^ cfu/mL. For infection, the PAO1culture was diluted to 1.0 × 10^7^ cfu/50 μL in PBS or PBS supplemented with 300 μM terrein. Mice were anesthetized before infection by intraperitoneal injection with Zoletil 50 (50 g/L) + Rompun (23.32 g/L) at 0.006 mL/10 g + 0.004 mL/10 g, respectively. Infections were conducted via the intranasal route by making each mouse inhale 50 μL of each bacterial suspension (n = 7 for each group). Infected mice were observed for survival for 24 h. Six mice, three per group, were euthanized at 9 h post-infection to count viable PAO1 cells in mouse lungs. The lungs were extracted and homogenized in 1 mL PBS. The viable count assay of each homogenate was conducted in triplicate.

### QS competition assay

An AHL-based QS competition assay was conducted using two genetically modified strains, *A*. *tumefaciens* NT1 and *C*. *violaceum* CV026^25^. *A*. *tumefaciens* NT1 or *C*. *violaceum* CV026 was cultivated in LB medium overnight at 220 rpm at 30 °C. Next, 1 mL of the 20-fold diluted overnight culture was dispensed into a 15- mL conical tubes, followed by addition of 5 μL X-gal (20 g/L) and 10 μL of 100 μM OdDHL (Sigma) to *A*. *tumefaciens* NT1 and 10 μL of 50 mM BHL (Cayman, Ann Arbor, MI, USA) to *C*. *violaceum* CV026. Ten microliters of each test compound dissolved in DMSO, or DMSO as a negative control, were added to the cultures and incubated at 30 °C for 48 h. QS inhibition was assayed by measuring the OD at 590 nm and 545 nm for *C*. *violaceum* CV026 and *A*. *tumefaciens* NT1, respectively, while cell viability was assayed by measuring the OD at 600 nm using a microtiter ELISA reader.

### Measurement of QS signaling molecules and analysis of QS gene expression

The effects of terrein on the production of QS signaling molecules^55^ and the transcription of QS-regulated genes^56^ were determined as previously described. Overnight cultures of *P*. *aeruginosa* PAO1 or PA14 were diluted 100–fold in LB medium, and 3 mL of culture was dispensed into 50-mL conical tubes, treated with test compounds dissolved in DMSO, and incubated at 220 rpm at 37 °C for 24 h. The cultures were then centrifuged at 12,000 rpm at 4 °C for 10 min. For the measurement of QS signaling molecules, the three QS signaling molecules, OdDHL, BHL, and PQS, were extracted from 2 mL of culture supernatants. Extraction was performed with 2 mL of ethyl acetate, which was acidified with 0.1% acetic acid, and the organic layer was evaporated under a vacuum and quantified by LC-MS/MS in MRM mode as described in the Supporting Information. For the analysis of QS gene expression, total RNA was extracted using TRIzol reagent (Invitrogen) according to the manufacturers’ instructions. cDNA for RT-qPCR detection of the expression of target genes was synthesized, followed by RT-qPCR using the Bio-Rad CFX-96 real time system (Bio-Rad, Hercules, CA, USA) with the primers listed in Table S1 as described in the Supporting Information. mRNA expression was normalized using the endogenous *rpoD* gene.

### Measurement of cellular c-di-GMP

C-di-GMP was analyzed using a previously described method^57^. To analyze the c-di-GMP in biofilm cells, overnight cultures of PAO1 or PA14 were diluted 100–fold in M63 medium and dispensed at 0.1 mL/well in 96-well polystyrene microtiter plates. Test compounds dissolved in DMSO or DMSO as a negative control were used to treat 10 wells. After forming biofilms in the wells at 37 °C without agitation for 9 h, the suspended cells were discarded, and the biofilms cells were washed with distilled water. Next, 0.1-mL of fresh M63 medium was added to each well, and the biofilms cells were detached via sonication for 2 min and collected. After centrifugation at 12,000 × g at 4 °C for 10 min, the harvested cells were resuspended in M63 medium. Then, 70% v/v perchloric acid (HClO_4_) was added to the resuspensions at a final concentration of 0.6 M. The samples were incubated initially on ice for 30 min, and all the subsequent steps performed at 4 °C. After centrifuging the samples at 12,000 g at 4 °C for 10 min, the supernatants were transferred to 1.5 mL-microfuge tubes. Protein in the precipitates was measured with the Pierce BCA protein assay (Thermo Scientific), which was used to normalize the amount of c-di-GMP. A 1/5 volume of 2.5 M KHCO_3_ (20 μL) was added to the nucleotide extracts to neutralize the pH and then briefly centrifuged to collect the samples. Then, the supernatants were transferred to fresh 1.5-mL microfuge tubes. After centrifuging for 10 min to remove the perchlorate salt precipitates, c-di-GMP in the supernatants was quantitatively analyzed using LC-MS/MS in MRM mode as described in the Supporting Information.

#### PDE and DGC activity assays

PDE^58^ and DGC^59^ activities in *P*. *aeruginosa* were examined as reported previously. Overnight cultures of *P*. *aeruginosa* PA14 were diluted 100–fold in 1 mL of LB medium, treated with the test compound, and incubated in a shaking incubator at 220 rpm and 37 °C for 9 h. After centrifugation of the cultures at 12,000 × g and 4 °C for 10 min, harvested cells were resuspended in 1 mL and 200 μL of buffer for the PDE and DGC assays, respectively. The buffer for the PDE assay contained 50 mM NaCl, 50 mM Tris base (pH 8.1), 1 mM MnCl_2_, and 5 mM bis-pNPP. Bis-pNPP (Sigma) was used as a synthetic substrate for c-di-GMP-specific PDE. The buffer for the DGC assay contained 75 mM Tris-HCl at pH 7.8, 250 mM NaCl, 25 mM KCl, and 10 mM MgSO_4_. The resuspended cells were lysed by sonication 10 times for 30 sec each. For the PDE reaction, the mixture was incubated at 37 °C for 2 h in a shaking incubator at 60 rpm. The degradation of bis-pNPP into p-nitrophenol was then measured at 410 nm using a microtiter ELISA reader. For the DGC reaction, the supernatants were obtained by centrifugation, and 25 μM GTP was added as the substrate. Then, the mixture was incubated at 37 °C for 2 h in a shaking incubator at 60 rpm. Cell lysates before GTP addition were used as a blank. The c-di-GMP amount produced from GTP by the DGC enzyme was measured by LC-MS/MS. PDE (OD at 410 nm) and DGC activities (c-di-GMP in pmol) were then normalized by total proteins measured using the Pierce BCA protein assay.

### Statistical analysis

Data are expressed as the means ± SD. The unpaired Student’s *t* test was used to analyze the data (Excel software, Microsoft, Redmond, WA, USA). A *p*-value < 0.05 was considered statistically significant.

### Ethics

All animal experiments were approved by the Committee on the Ethics of Animal Experiments of Yonsei University College of Medicine (IACUC permit number: 2013-0369-5). All experiments were performed in accordance with the approved guidelines of the Institutional Ethical Committee, adhered to Guide for the Care and Use of Laboratory Animals of National Research Council (USA).

## Electronic supplementary material

Supporting Information
