# Supplementary material for: Terrein is an inhibitor of quorum sensing and c-di-GMP in Pseudomonas aeruginosa: a connection between quorum sensing and c-di-GMP
Source: Sci Rep. 2018 Jun 5;8:8617. doi: 10.1038/s41598-018-26974-5 (PMC5988783; doi:10.1038/s41598-018-26974-5)
Supplement: Supplementary file 1 — Supporting Information [file 41598_2018_26974_MOESM1_ESM.pdf]

Supporting Information

Terrein is an inhibitor of quorum sensing and c-di-GMP in *Pseudomonas aeruginosa*: a connection between quorum sensing and c-di-GMP

Bomin Kim[1], Ji-Su Park[1], Ha-Young Choi[1], Sang Sun Yoon[2], and Won-Gon Kim[1]

[1]Superbacteria Research Center, Korea Research Institute of Bioscience and Biotechnology, Yusong, Daejeon 305-806, Korea. [2]Department of Microbiology and Immunology, Yonsei University College of Medicine, Seoul03722, Korea. Bomin Kim and Ji-Su Park contributed equally to this work. Correspondence and requests for materials should be addressed to W.-G. K. (e-mail: wgkim@kribb.re.kr).

Isolation of terrein. Fermentation of the producing strain, *Aspergillus terreus* FN423, was carried out in a liquid culture medium containing Potato Dextrose Broth (Becton, Dickinson and Company, Franklin Lakes, NJ, USA). For the production of an active compound, 5 ml of the seed culture were transferred into 500 ml Erlenmeyer flasks containing 100 ml of the above sterile medium, and cultivated on a rotary shaker (150 rpm) at 28℃ for 10 days. After centrifugation (4000 rpm, 10 min) of the cultures, the supernatants (3 L) were extracted with an equal volume of ethyl acetate (EtOAc). The crude EtOAc extract (1.58 g) was subjected to SiO_2 column chromatography followed by stepwise elution with chloroform ($CHCl_3$) - methanol (MeOH) (30:1 ~ 5:1). The active fractions eluted with $CHCl_3$-MeOH (20:1) were pooled and concentrated *in vacuo*. The residue (849 mg) was applied again to a Sephadex LH-20 and then eluted with MeOH to yield terrein (285 mg). The purity of the isolated terrein was determined to be over 99% at 220 nm by analytical HPLC (Fig. S1a).

NMR, MS, and optical rotation data of the isolated terrein

Terrein: $[\alpha]_D$ = +129.196.7° (*c* = 0.1, H_2O); ESI-MS: *m/z* 177 $(M+Na)^+$; ^{1}H-NMR (400 MHz, CD_3OD+$CDCl_3$): 6.80 (1H, dq, *J* = 15.8 and 6.8 Hz, H-7), 6.32 (1H, d, *J* = 15.8 Hz, H-6), 5.88 (1H, s, H-5), 4.66 (1H, d, *J* = 2.5, H-3), 4.10 (1H, d, *J* = 2.5, H-2), 1.86 (1H, d, J = 6.8 Hz, H-8), ^{13}C-NMR (100 MHz, CD_3OD): 205.8 (C-1), 171.0 (C-4), 142.0 (C-7), 126.62 (C-6), 126.1 (C-5), 82.6 (C-2), 78.3 (C-3), 19.6 (C-8).

Acetylation of terrein. Acetic anhydride (1 mL) was added to a solution of terrein (10 mg) in pyridine (1 mL), and the mixture was stirred at 25°C for 3 h, after which time the pyridine and excess acetic anhydride were evaporated under a vacuum. The residue was partitioned with EtOAC, and the resultant EtOAc extract was purified by SiO_2TLC developed with Hexane-EtOAc (2:1) to give diacetyl terrein (8.5 mg) at an R_f of 0.16. The structure of diacetyl terrein was confirmed by NMR and MS data.

Diacetyl terrein: a yellowish powder. ESI-MS: *m/z* 219 $[M+H]^+$, 261 $[M+Na]^+$; ^{1}H-NMR (400 MHz, $CDCl_3$): 6.38 (1H, dq, *J* = 16.0 and 6.4 Hz, H-7), 6.31 (1H, d, *J* = 16.0 Hz, H-6), 6.19 (1H, s, H-5), 6.08 (1H, brs, H-3), 5.22 (1H, d, *J* = 2.0 Hz, H-2), 1.94 (1H, d, *J* = 6.4 Hz, H-8), 2.16 (3H, s, $O(C=O)CH_3$), 2.15 (3H, s, $O(C=O)CH_3$) ^{13}C-NMR (125 MHz, $CDCl_3$): 197.1 (C-1), 170.4 ($O(\underline{C}=O)CH_3$), 170.3 ($O(\underline{C}=O)CH_3$), 165.3 (C-4), 140.4 (C-7), 124.7 (C-6), 128.3 (C-5), 78.4 (C-2), 74.9 (C-3), 21.0($O(C=O)\underline{C}H_3$), 20.7($O(C=O)\underline{C}H_3$), 19.7 (C-8).

Pyocyanin and rhamnolipid assay. For both pyocyanin and rhamnolipid production, after overnight cultures of *P. aeruginosa* PAO1 were diluted 100–fold in LB medium, 5 mL of culture was dispensed into 50 mL conical tubes, treated with test compounds dissolved in DMSO, and incubated at 220 rpm at 37°C for 24 h. The cultures were then centrifuged at 12,000 rpm at 4°C for 10 min. For pyocyanin assay, 5 mL of supernatant was mixed with 3 mL of chloroform and 2 mL of 0.2 N HCl was added to the chloroform fraction. The resultant aqueous fraction was measured at 520 nm by a microplate reader. For rhamonolipid assay, 500 μL supernatant was mixed with 500 μL diethyl ether. The ether fraction was evaporated to dryness and dissolved in 500 μL deionized water. 100 μL of the water extract was mixed with 900 μL of Orcinol solution (0.19% Orcinol (Sigma) in 53% H_2SO_4). The mixture was boiled for 30 min, cooled at room temperature for 15 min, and then measured at 421 nm by a microplate reader.

Biofilm assay. Overnight cultures of *P. aeruginosa* PAO1 were diluted 100–fold in M63 medium and the dilutions were dispensed at 0.1 mL/well in a 96-well polystyrene microplate. Test compounds or DMSO as a negative control were added to the wells. After incubation at 37°C for 9 h without agitation, the unattached cells and media were removed and the cells forming the biofilm, which remained attached to the well's surface, were stained using 120 μL of 0.1% crystal violet for 10 min. The bound crystal violet was solubilized with 150 μL of 30% acetic acid in water for 15 min. The OD of the eluted crystal violet was measured at 550 nm by a microplate reader.

Confocal laser scanning microscopy. Biofilms of PAO1 were grown on 15 mm^2 glass coverslips (Matsunami Glass Ind., Ltd. Japan). Sterile coverslips were positioned vertically in 24-well plates. Cultures were grown for 6 h without agitation at 37°C. Coverslips were then washed and stained with SYTO9/propidium iodide according to the manufacturer′s instructions of the L13152 LIVE/DEAD Baclight bacterial viability kit (Invitrogen Molecular Probes, USA). After staining for 15 min in the dark, biofilms were washed with sterile phosphate-buffered saline (PBS) to remove the planktonic dyes and bacteria, and then biofilms were visualized by excitation with an argon laser at 488 nm (emission: 515 nm) and 543 nm

(emission: 600 nm) under a Confocal laser scanning microscope (Carl Zeiss LSM800, Jena, Germany).

Quantification of QS signaling molecules by LC-MS/MS

The samples were subjected to an HPLC system (Luna C18(2), 100 × 2.0 mm, 3 μm, Phenomenex, Torrance, CA, USA) connected to a QTrap 3200 with a TurboIon Spray source (AB SCIEX, Singapore). The column was maintained at 20°C with a flow rate of 0.4 mL/min and a gradient of acetonitrile in 0.1% (v/v) aqueous formic acid; 0-10 min from 70% to 100%. MRM was performed by selecting the two mass ions set specifically for the selected analytes to detect the transition from parent ion to product ion, i.e., *m/z* 298.211 > 197.200 for OdDHL, *m/z* 172.181 > 71.000 for BHL, and *m/z* 260.244 > 188.100 for PQS (Sigma). For analysis of OdDHL, BHL, and PQS, the TurboIon Spray source-dependent parameters were optimized to the following values: 10 psi curtain gas, high collision gas, 5500 V ion spray voltage, 400°C temperature, and 12 psi ion source gas. The compound-dependent parameters for OdDHL, BHL, and PQS were optimized to the following values: 19, 17, and 41 eV collision energy; 5, 8, and 12 V entrance potential; 276, 31, and 71 V declustering potential; and 18, 10, and 16 V collision cell exit potential, respectively.

Quantification of c-di-GMP by LC-MS/MS

The samples were subjected to an HPLC system (Luna C18(2), 100 × 2.0 mm, 3 μm, Phenomenex, Torrance, CA, USA) connected to a QTrap 3200 with a TurboIon Spray source (AB SCIEX, Singapore). The column was maintained at 20°C with a flow rate of 0.4 mL/min and a gradient of acetonitrile in 0.1% (v/v) aqueous formic acid; 0-5.6 min from 2% to 30% and 5.6-7 min from 30% to 80%. MRM was performed by selecting the following mass ions: *m/z* 691.058 > 152.200 for c-di-GMP. For analysis of c-di-GMP, the optimized TurboIon Spray source-dependent parameters were as follows: 10 psi curtain gas, medium collision gas, 5500 V ion spray voltage, 400°C temperature, and 12 psi ion source gas. The compound-dependent parameters were optimized to the following values: 49 eV collision energy, 8.5 V entrance

potential, 81 V declustering potential, and 4 V collision cell exit potential.

RT-qPCR of QS-regulated genes

cDNA was synthesized from 2 μg of RNA mixed with 1 μg of random primers (Promega C1181) and RNase-free water (Sigma W4502) in a total of 13.37 μL and incubated for 5 min at 70°C. Thereafter, 1 μL of M-MLV reverse transcriptase (Promega M170 200 U/μL), 5 μL 5 × M-MLV buffer (Promega M531), 5 μL of deoxynucleoside triphosphates (Enzynomics N001S, 2 mM), and 0.63 μL of RNasin® Ribonuclease Inhibitors (Promega N251, 2500 U/μL) were added and incubated for 60 min at 42°C. The cDNA samples were used for RT-qPCR detection of the expression of target genes. RT-qPCR was performed using the Bio-Rad CFX-96 real time system (Bio-Rad, Hercules, CA, USA) with the primers listed in Table S1. Amplification and expression were carried out in a total volume of 20 μL containing 10 μL SYBR Premix Ex Taq™ (Takara, Shiga, Japan), 1 μL each of the forward and reverse primers (5 μM) of target genes, 2 μL template cDNA, and 6 μL RNase-free water. The cycling parameters were as follows: initial activation at 95℃ for 30 s; 40 cycles at 95℃ for 5 s, 60℃ for 30 s, and melting curve analysis at 95℃ for 15 s, 60℃ for 5 s and 95℃ for 5 s. mRNA expression was normalized using the endogenous *rpoD* gene.

Table S1. Primers used for quantitative RT-PCR

Gene and primer type	PCR primer sequence (5' to 3')
lasI	
Forward	TTCAAGGAGCGCAAAGGCTG
Reverse	GTTCTTCAGCATGTAGGGGC
lasR	
Forward	TCTGGGAACCGTCCATCTAC
Reverse	GACCGACTCCATGAAACGGT
lasA	
Forward	GACGACCTGTTCCTCTACGG
Reverse	GCTCCAGGTATTCGCTCTTG
lasB	
Forward	CCGCAAGACCGAGAATGACA
Reverse	CTTCCCACTGATCGAGCACT
aprE	
Forward	ATGTACATCGTGCCCAACAG
Reverse	GGTCTTGCTCTGGTTGAAGG
rhlI	
Forward	CTTCATCGAGAAGCTGGGCT
Reverse	AGGTAGGCGAAGACGTCCTT
rhlR	
Forward	TGCATGATCGAGTTGCTG
Reverse	GTGCTCTCGGAGATGCTCA
rhlA	
Forward	GCGCGAAAGTCTGTTGGTAT
Reverse	CAGCACCACGTTGAAATGTT
pqsA	
Forward	CCACTCCGCTGGACGACAAC
Reverse	GCAGCATGTGCGAGGGAATC
phnB	
Forward	CACTCGCTGGTGGTCAGTC
Reverse	AGAGTAGAGCGTTCTCCAGCA
pqsH	
Forward	ATGTCTACGCGACCCTGAAG
Reverse	AACTCCTCGAGGTCGTTGTG
pqsR	
Forward	CTTCGCCTGATCCCTTACAT
Reverse	TGAAATCGTCGAGCAGTACG
phzA2	
Forward	AACCACTTCTGGGTCGAGTG
Reverse	TCGAGTTCGAAGGAATGGAT
rpoD	
Forward	GGGGATCAACGTATTCGAGA
Reverse	GGTACCCATTTCACGCATGT

a

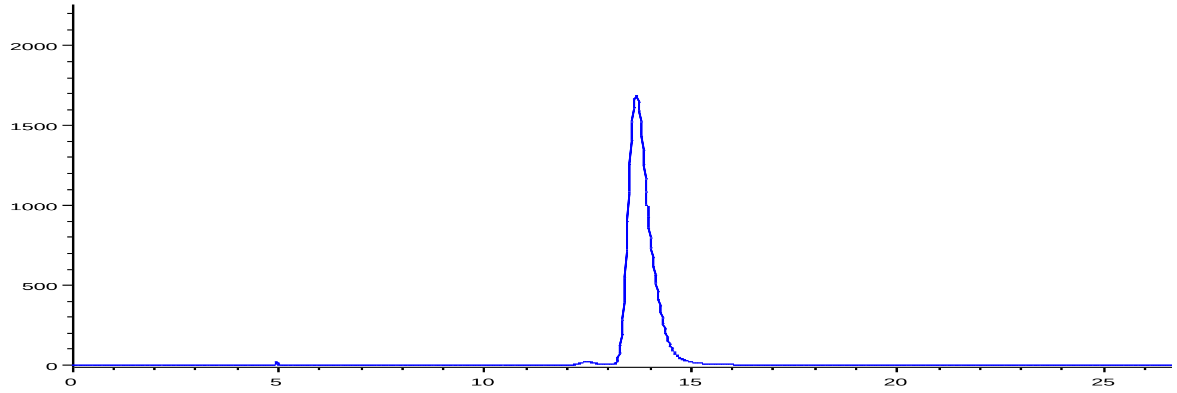

b

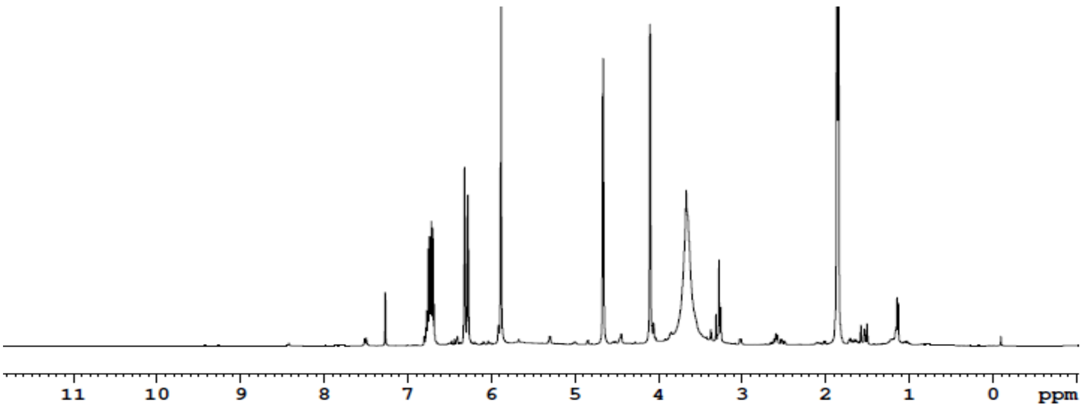

c

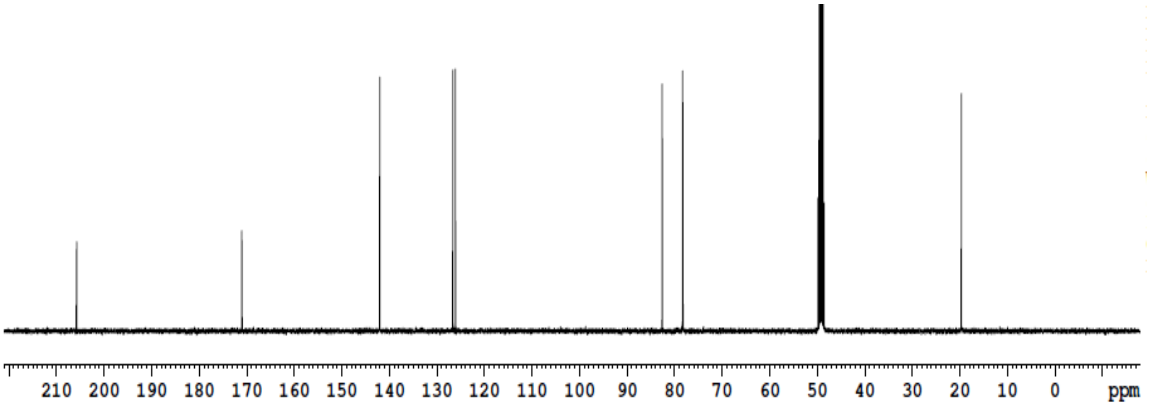

Supplementary Figure S1. HPLC profile and NMR spectral analysis of terrein isolated from *Aspergillus terreus* FN423. (a) The purity of the isolated compound was determined to be over 99% at 220 nm by analytical HPLC (column 4.6 x 250 mm, S-4 μm, YMC C_{18}) with 5:95

acetonitrile/water at a flow rate of 0.8 mL/min and a retention time of 13.8 min. (b) ^{1}H NMR spectrum (400 MHz, CD_3OD+$CDCl_3$). (c) ^{13}C NMR spectrum (100 MHz, CD_3OD+$CDCl_3$).

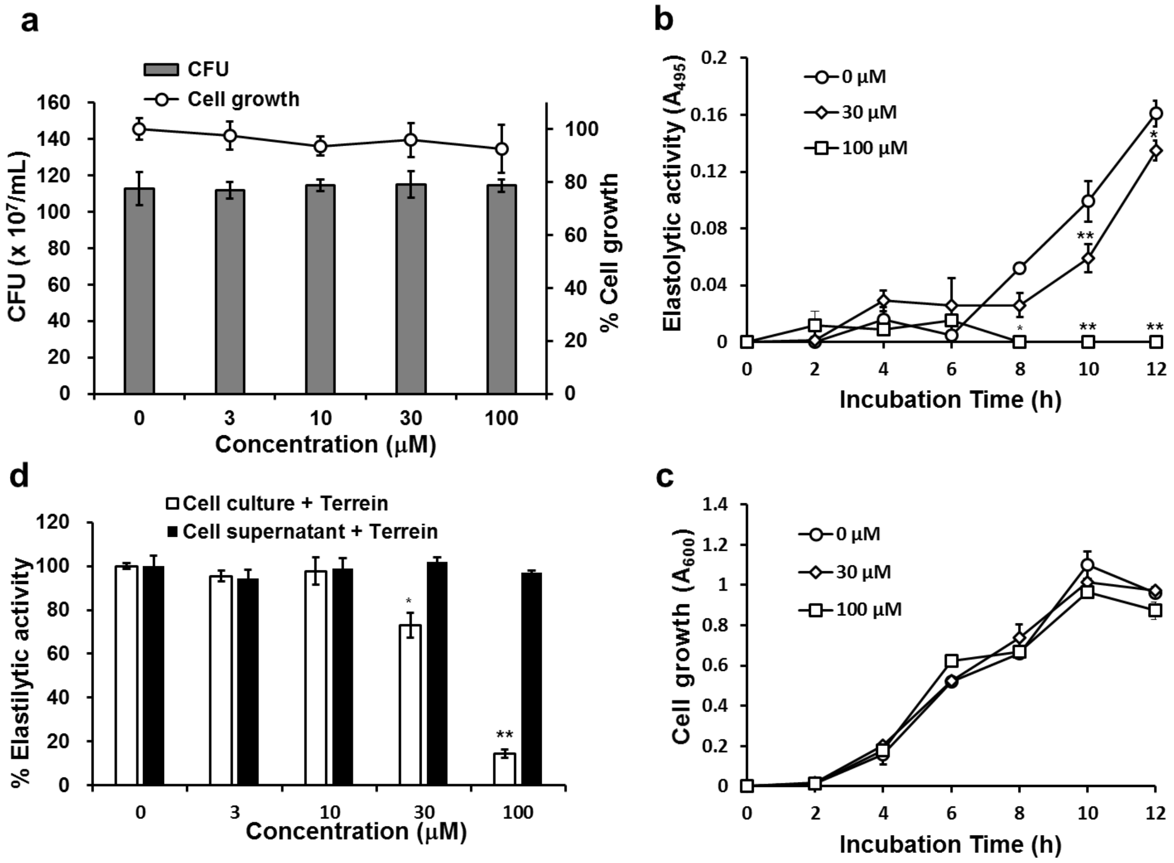

Supplementary Figure S2. Effects of terrein on cell viability, growth-dependent elastase production, and elastase activity. (a) Dose-dependent effect of terrein on bacterial growth. The cell density was measured at 600 nm and the number of viable cells was counted in each culture after 18 h of growth. Three independent experiments were performed, and the mean ± SD values are displayed in each bar. (b) Growth-dependent effect of terrein on elastase production. PAO1 cells were grown in LB medium containing two different concentrations of terrein for different times, and culture supernatants were harvested at the given time points to measure elastase activity. Each experiment was repeated in triplicate, and the mean ± SD values are displayed. *, $P < 0.05$; **, $P < 0.01$ versus elastase activity from untreated PAO1 cells. (c) Growth curves of PAO1 cells grown aerobically in shaking LB medium and LB medium with terrein. Each growth curve experiment was repeated in triplicate, and the mean ± SD values are displayed. (d) Dose-dependent effect of terrein on elastase activity. The PAO1 cells was grown in LB medium containing various concentrations of terrein for 18 h, and culture supernatants were harvested to measure elastase activity. Aliquots of PAO1 culture supernatants derived from 18 h of culture in LB medium were treated with various concentrations of terrein for 7 h and assayed for elastase activity. Two independent experiments in triplicate were carried out, and the mean ± SD values are displayed in each bar. *, $P < 0.001$; **, $P < 0.0001$ versus elastase activity from untreated PAO1 cells.

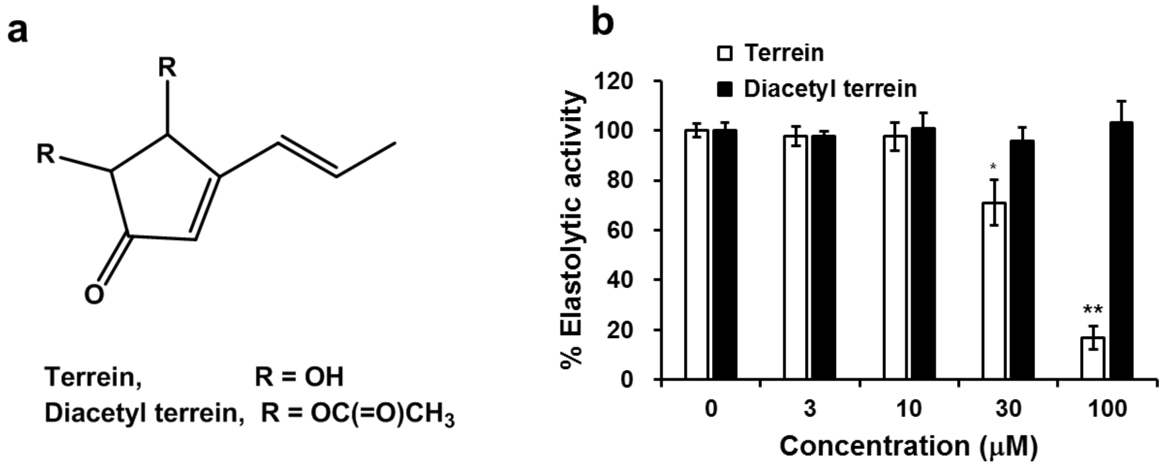

Supplementary Figure S3. Chemical structure of diacetyl terrein (a) and comparison of terrein and diacetyl terrein on elastase activity (b). The PAO1 cells was grown in LB medium containing various concentrations of terrein or diacetyl terrein for 18 h, and then elastase activity was assayed from the culture supernatants. . Two independent experiments in triplicate were carried out, and the mean ± SD values are displayed in each bar. *, $P < 0.001$; **, $P < 0.0001$ versus elastase activity from untreated PAO1 cells.

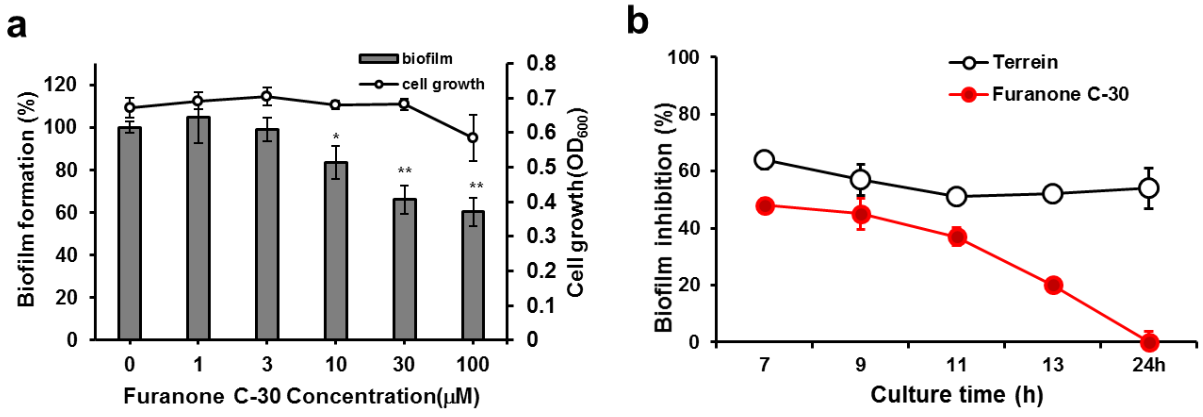

Supplementary Figure S4. Anti-biofilm activity of furanone C-30 and comparison of biofilm-inhibitory activity of terrein and furanone C-30 depending on the culture time. (a) *P. aeruginosa* PAO1 biofilm was formed in the presence of furanone C-30 for 9 h. The biofilm cells attached to the well surface were assayed using crystal violet staining. (b) Biofilm formation of *P. aeruginosa* PAO1 cultured with terrein (100 μM) or furanone C-30 (100 μM) was assayed at the different culture time (7 – 24 h). Two independent experiments in triplicate were carried out, and the mean ± SD values are displayed in each bar. *, $P < 0.001$; **, $P < 0.0001$ versus elastase activity from untreated PAO1 cells.

.

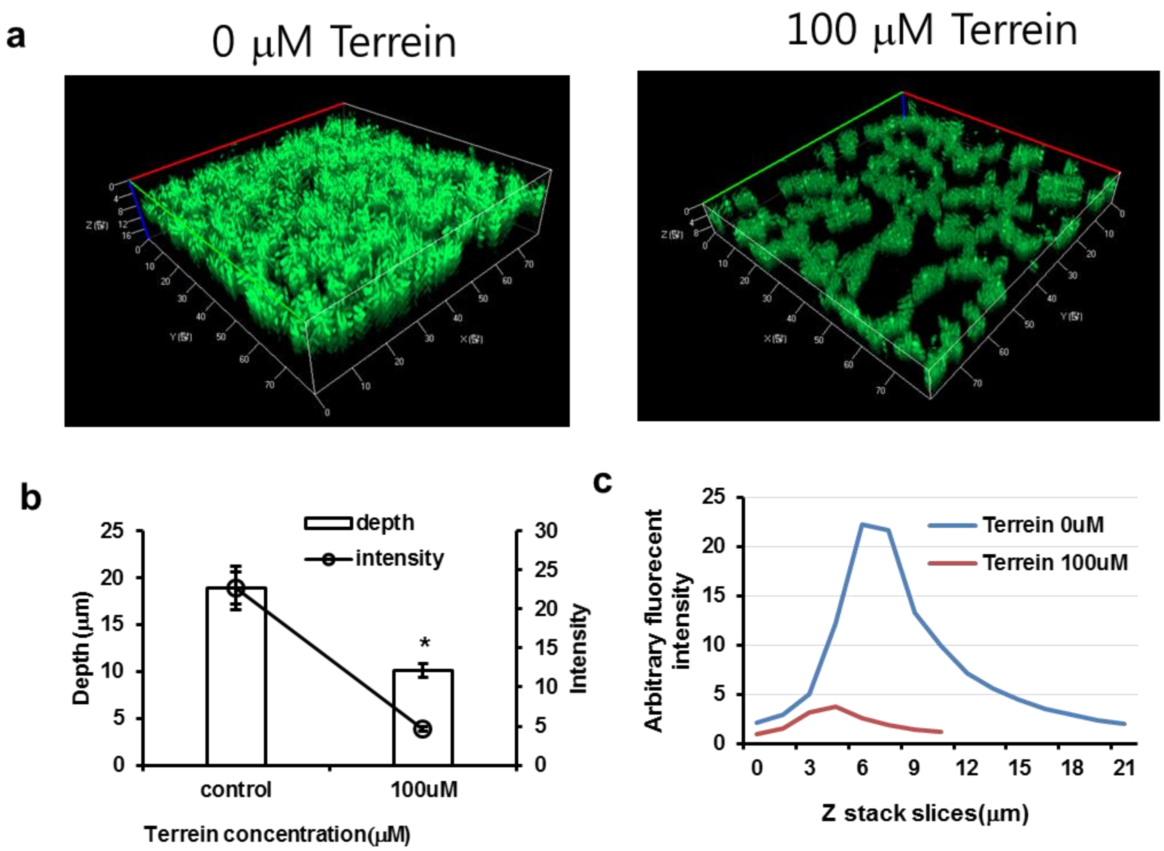

Supplementary Figure S5. Confocal laser scanning microscope analyses of *P. aeruginosa* biofilms treated with terrein. (a) A POA1 strain was grown on glass coverslips in a 24-well plate for 6 h in the medium containing 0 μM or 100 μM terrein. Biofilms were stained with the BacLight Live/Dead Viability Kit. Cells staining green are viable cells. The experiments were performed twice and representative images are shown. The scale bar represents 20 μM. (b) Biofilm thickness and quantification of green fluorescent intensities of two biofilms. Data represent the average of image stacks collected from five randomly selected areas. *, $P < 0.0001$ versus biofilm from untreated PAO1 cells. (c) The green fluorescent intensities in each of the sliced focal planes taken at 1.5 μm intervals are plotted as a function of biofilm height and compared between two biofilms.

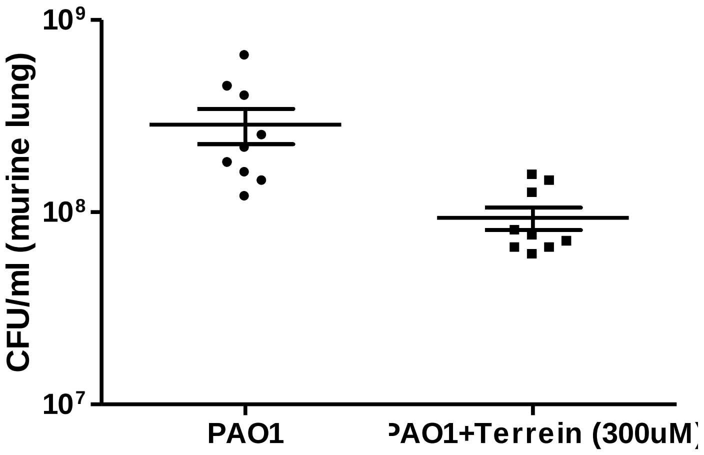

Supplementary Figure S6. Inhibition of *P. aeruginosa* PAO1 virulence by terrein in murine airway infection model. Inhibition of PAO1 virulence by terrein in the murine airway infection model. Seven mice were used in each group, and the infection dose was 1×10^7 cfu. Bacterial counts were recovered from infected mouse lungs. *P*=0.0059 versus the cfu from mice that were not treated with terrein.

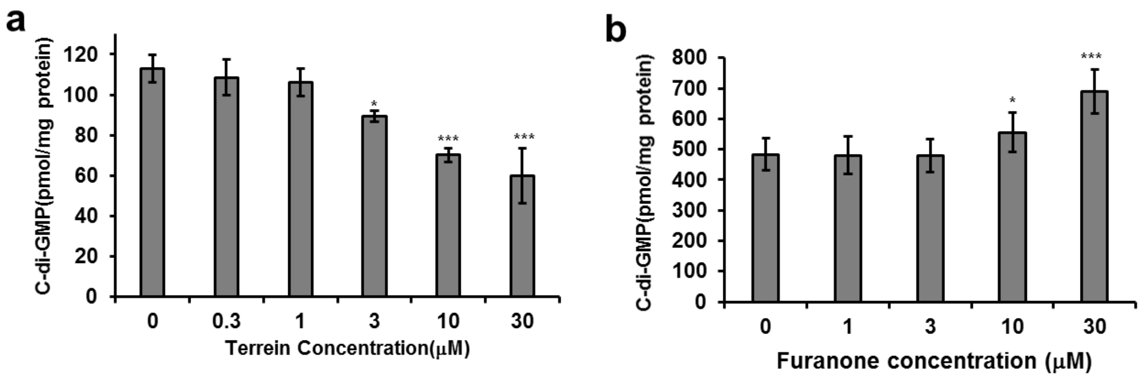

Supplementary Figure S7. Effects of terrein on cellular c-di-GMP. Cellular c-di-GMP levels in the biofilm cells of PAO1 cultured with different terrein or furanone C-30 concentrations for 9 h. After the biofilms were dissociated from the wells by gentle sonication, cellular c-di-GMP was extracted from the biofilm cells, measured, and normalized by total proteins. The experiment shown is representative of three independent experiments in triplicate, and the mean ± SD values are displayed in each bar. *, $P < 0.01$; **, $P < 0.001$; ***, $P < 0.0001$ versus DMSO treatment.

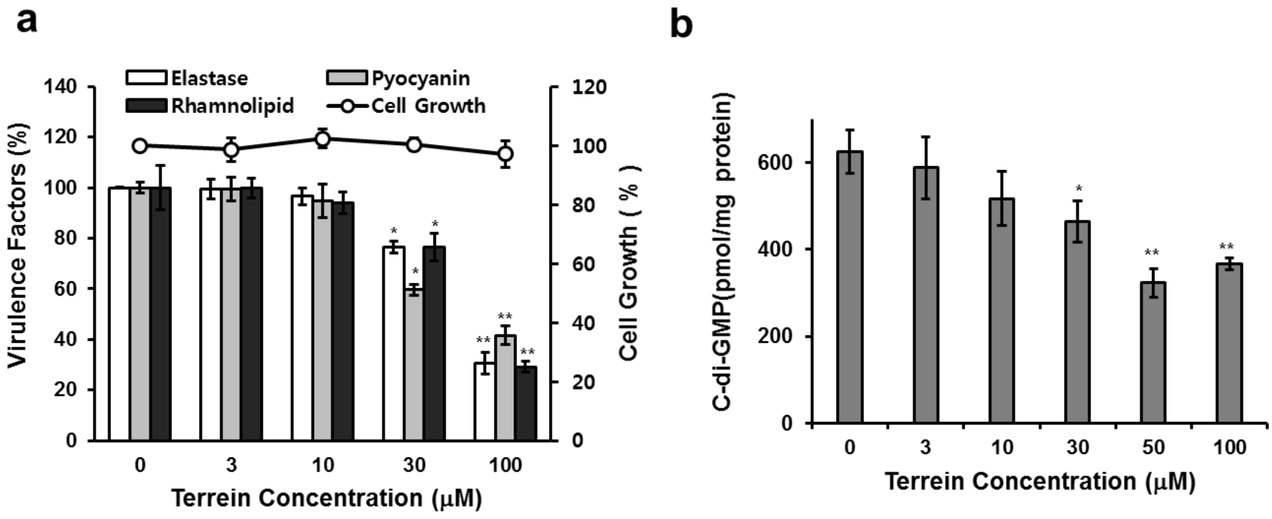

Supplementary Figure S8. Effects of terrein on virulentce factor production and on c-di-GMP levels in *P. aeruginosa* PA14. (**a**) Effects of terrein on virulence factor production and cell viability in *P. aeruginosa* PAO1. PA14 cells were grown in LB medium containing various concentrations of terrein for 24 h, and after cell density measurement at 600 nm, elasetase activity, pyocyanin, and rhamnolipid were measured in the culture supernatants. (b) Cellular c-di-GMP levels in the biofilm cells of PA14 cultured with different terrein or furanone C-30 concentrations for 9 h. After the biofilms were dissociated from the wells by gentle sonication, cellular c-di-GMP was extracted from the biofilm cells, measured, and normalized by total proteins. The experiment shown is representative of two independent experiments in triplicate, and the mean ± SD values are displayed in each bar. *, $P < 0.01$; **, $P < 0.001$; ***, $P < 0.0001$ versus DMSO treatment.

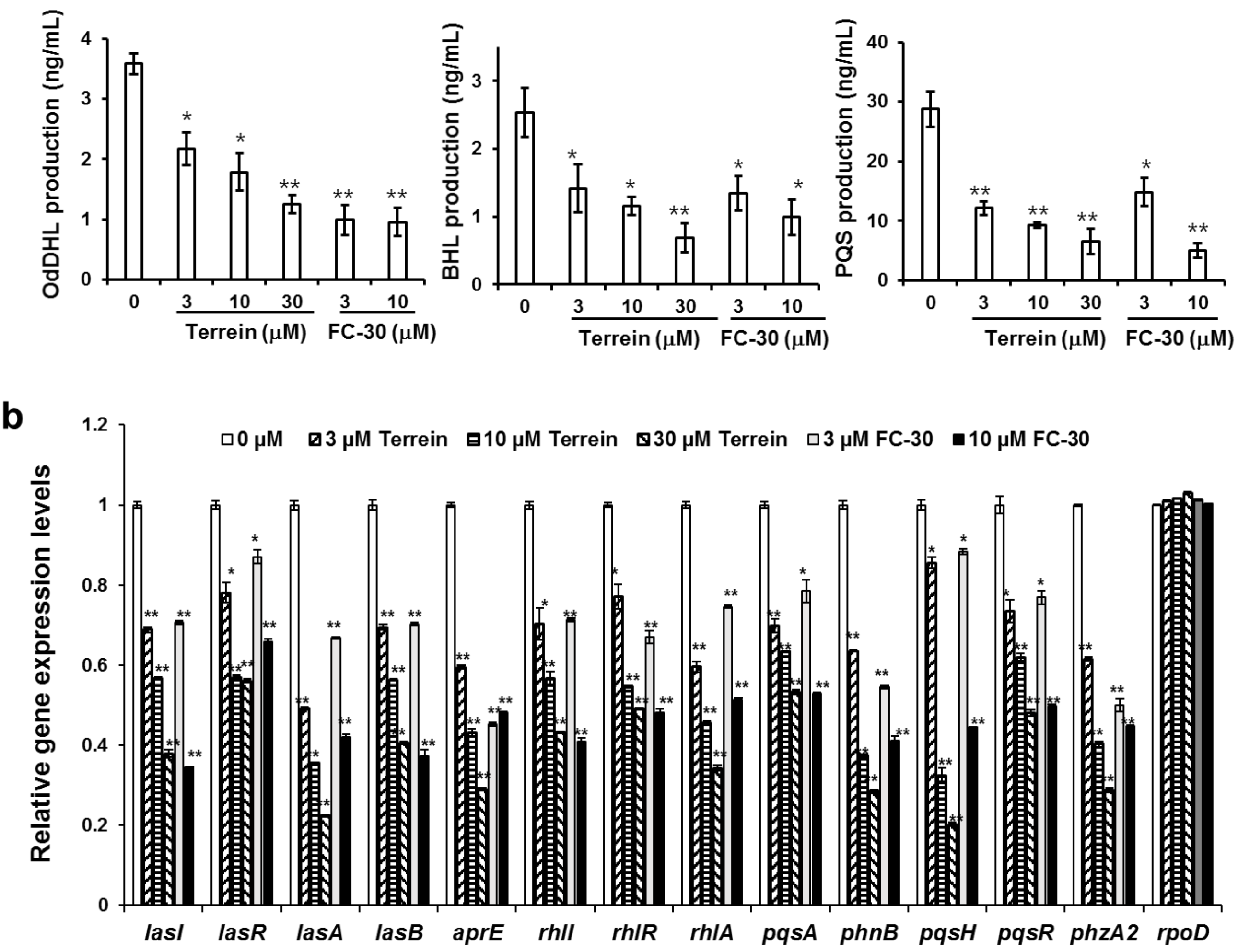

Supplementary Figure S9. Effect of terrein on the expression of QS-regulated genes in *P. aeruginosa* PA14. PA14 were cultured in LB medium containing with different terrein or furanone C-30 concentrations for 24 h. (**a**) Effect of terrein on the three main QS molecules, OdDHL, BHL, and PQS. The QS molecules were extracted from the culture supernatants and quantitatively analyzed by LC-MS/MS. The experiment shown is representative of three independent experiments in triplicate, and the mean ± SD values are displayed in each bar. *, $P < 0.01$; **, $P < 0.001$; ***, $P < 0.0001$ versus DMSO treatment. (**b**) Effect of terrein on the expression of QS-regulated genes as assessed by RT-qPCR. The experiment shown is representative of three independent experiments in triplicate, and the mean ± SD values are displayed in each bar. *, $P < 0.001$; **, $P < 0.0001$ versus DMSO treatment.

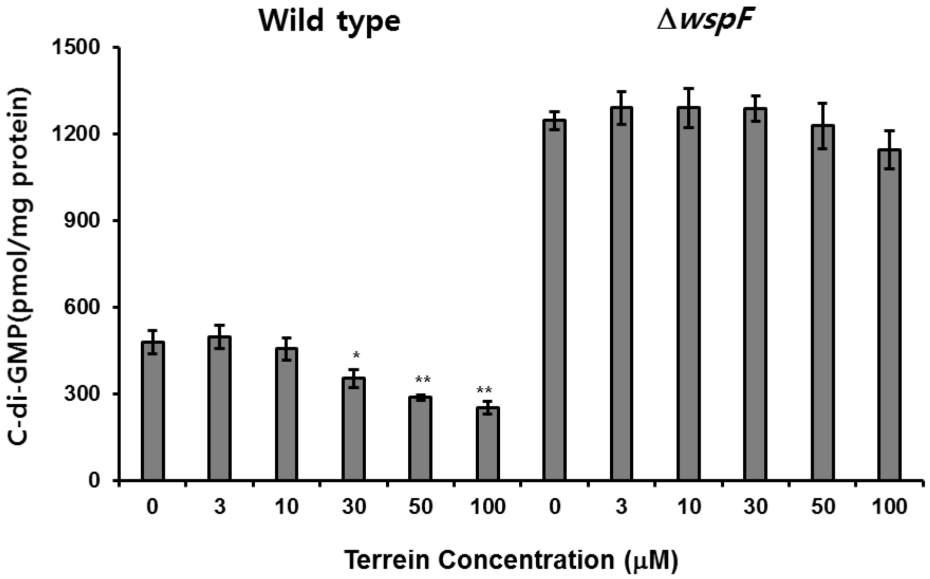

Supplementary Figure S10. Effects of terrein on cellular c-di-GMP of *P. aeruginosa* wspF mutant. Cellular c-di-GMP levels in the biofilm cells of *P. aeruginosa* wspF mutant versus wild-type PA14 cells cultured with different terrein for 9 h. After the biofilms were dissociated from the wells by gentle sonication, cellular c-di-GMP was extracted from the biofilm cells, measured, and normalized by total proteins. The experiment shown is representative of three independent experiments in triplicate, and the mean ± SD values are displayed in each bar. *, $P < 0.01$; **, $P < 0.001$ versus DMSO treatment.

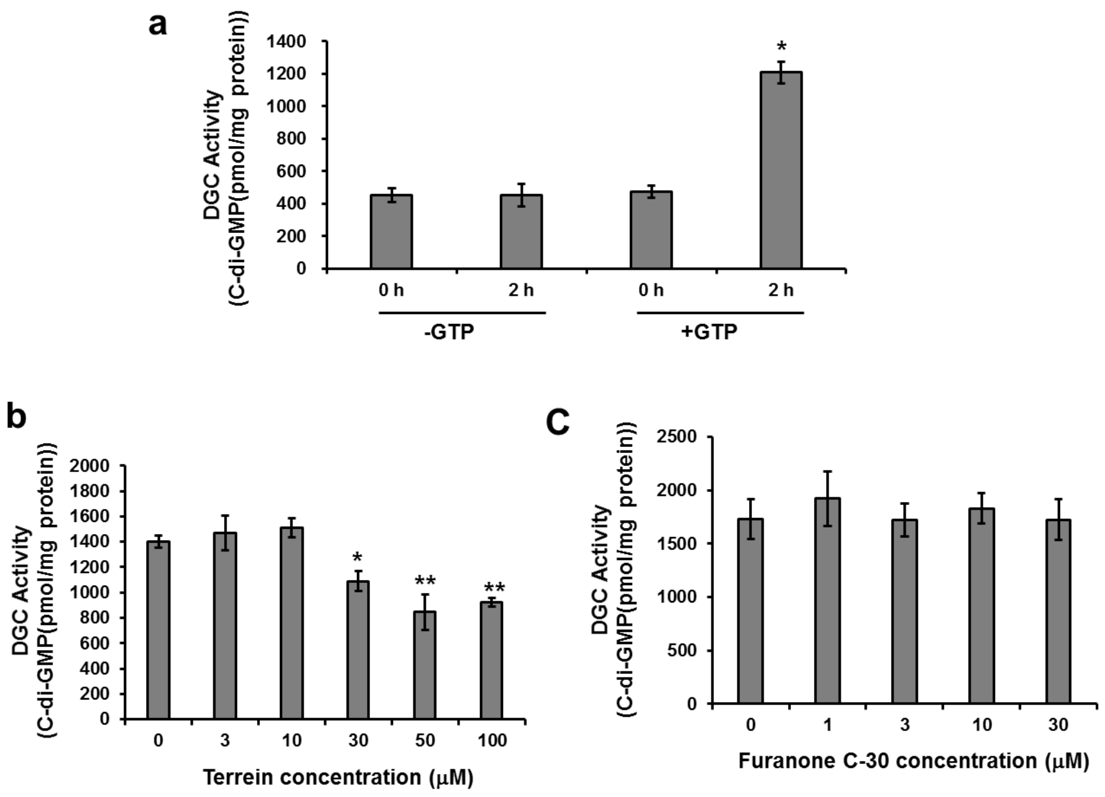

Supplementary Figure S11. Validation of the DGC assay and effects of terrein on DGC activity. (**a**) Analysis of natural c-di-GMP levels in the cell lysates for validation of the DGC assay. The natural c-di-GMP levels in the cell lysates of PAO1 cells cultured for 6 h were measured before and after the DGC assay of 2 h without or with GTP as a substrate. The experiment shown is representative of three independent experiments in triplicate, and the mean ± SD values are displayed in each bar. *, $P < 0.00001$ versus 0-h reaction. (**b** and **C**) The actuals levels of c-di-GMP produced by PA14 cells cultured with terrein or furanone C-30 for 6 h. Three independent experiments were performed in triplicate, and the mean ± SD values are displayed in each bar. *, $P < 0.001$; **, $P < 0.0001$; ***, $P < 0.00001$ versus untreated cells.

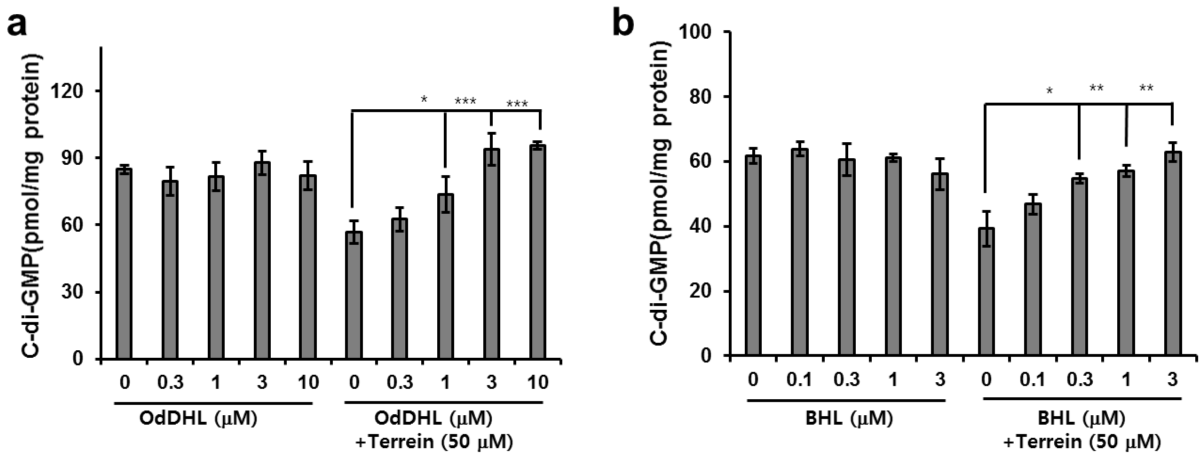

Supplementary Figure S12. Effects of exogenous QS ligands on the inhibition of cellular c-di-GMP by terrein. The actual c-di-GMP concentrations of PA14 cells cultured with terrein (50 μM) in the presence or absence of a different concentration of OdDHL (**a**) or BHL (**b**) for 9 h. Three independent experiments were performed in triplicate, and the mean ± SD values are displayed in each bar. *, $P < 0.01$; **, $P < 0.001$; ***, $P < 0.0001$ versus terrein-treated cells.

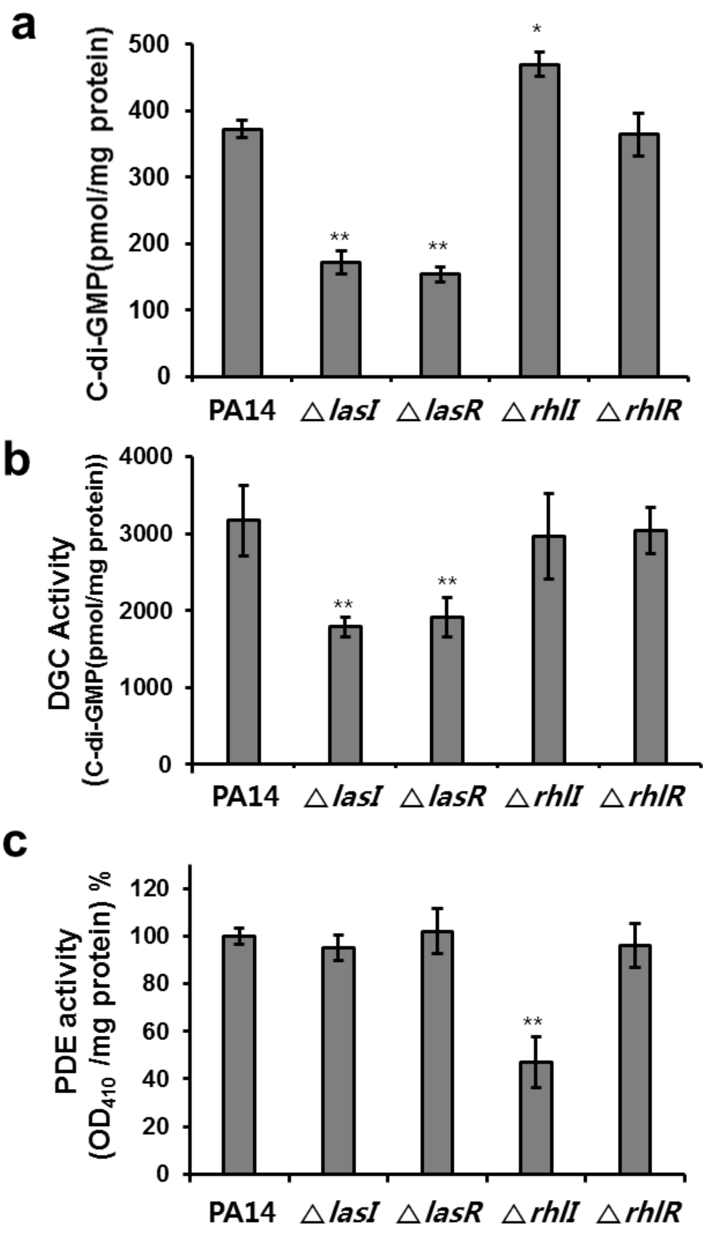

Supplementary Figure S13. C-di-GMP levels and c-di-GMP metabolizing enzymes in *P. aeruginosa* QS mutants. (**a**) The actual levels of cellular c-di-GMP in biofilm cells of *P. aeruginosa* QS mutants (*lasI*, *lasR*, *rhlI*, *rhlR* mutants) compared to those in wild-type PA14. (**b** and **c**) Production of c-di-GMP and degradation of the PDE-specific substrate bis-pNPP by *P. aeruginosa* QS mutants compared to wild-type PA14. Three independent experiments were performed in triplicate, and the mean ± SD values are displayed in each bar. *, $P < 0.001$; **, $P < 0.0001$ versus wild-type PA14.

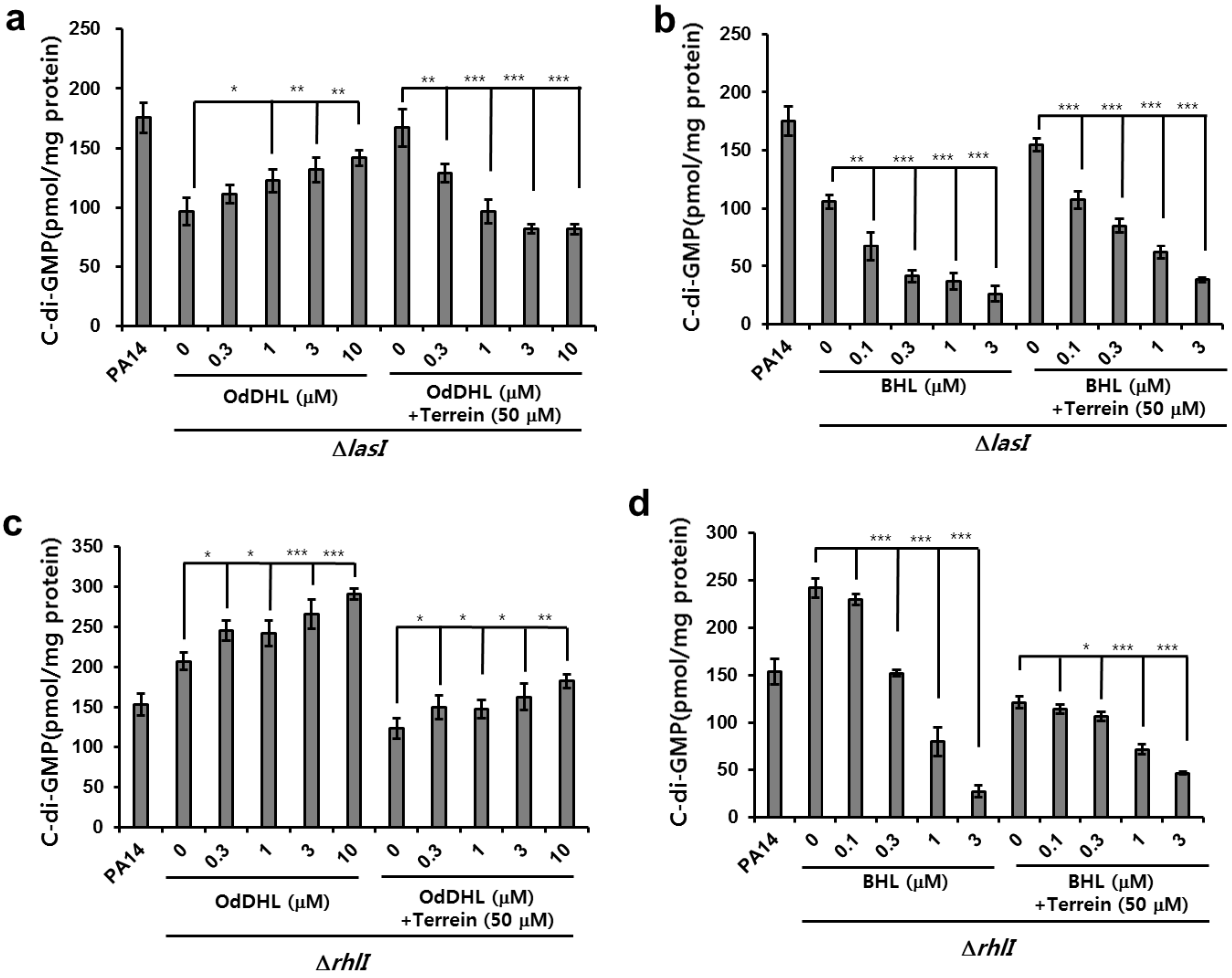

Supplementary Figure S14. Effects of addition of QS ligands on cellular c-di-GMP levels of *P. aeruginosa* QS mutants (*lasI* and *rhlI* mutants) and on the terrein's effects on cellular c-di-GMP levels of the mutants. (**a** and **b**) The actual c-di-GMP concentrations of *lasI* mutant cultured with terrein (50 μM) in the presence or absence of a different concentration of OdDHL (**a**) or BHL (**b**) for 9 h, compared to those in wild-type PA14. (**c** and **d**) The actual c-di-GMP concentrations of *rhlI* mutant cultured with terrein (50 μM) in the presence or absence of a different concentration of OdDHL (**c**) or BHL (**d**) for 9 h, compared to those in wild-type PA14. The experiment shown is representative of three independent experiments in triplicate, and the mean ± SD values are displayed in each bar. *, $P < 0.05$; **, $P < 0.01$; ; **, $P < 0.001$ versus DMSO treatment.
